# The mycobiome as integral part of the gut microbiome: crucial role of symbiotic fungi in health and disease

**DOI:** 10.1080/19490976.2024.2440111

**Published:** 2024-12-15

**Authors:** Hui Huang, Qiurong Wang, Ying Yang, Wei Zhong, Feng He, Jun Li

**Affiliations:** a Department of Clinical Medicine, Chengdu Medical College, Chengdu, Sichuan, P. R. China; b Department of Gastroenterology, First Affiliated Hospital of Chengdu Medical College, Chengdu, Sichuan, P. R. China; c Department of Gastroenterology, Sichuan Fifth People’s Hospital, Chengdu, China

**Keywords:** Gut mycobiome, commensal fungi, fungal dysbiosis, fungal‒bacterial interaction, immune response

## Abstract

The gut mycobiome significantly affects host health and immunity. However, most studies have focused on symbiotic bacteria in the gut microbiome, whereas less attention has been given to symbiotic fungi. Although fungi constitute only 0.01%–0.1% of the gut microbiome, their larger size and unique immunoregulatory functions make them significant. Factors like diet, antimicrobials use, and age can disrupt the fungal community, leading to dysbiosis. Fungal-bacterial-host immune interactions are critical in maintaining gut homeostasis, with fungi playing a role in mediating immune responses such as Th17 cell activation. This review highlights methods for studying gut fungi, the composition and influencing factors of the gut mycobiome, and its potential in therapeutic interventions for intestinal and hepatic diseases. We aim to provide new insights into the underexplored role of gut fungi in human health.

## 1. Introduction

The gut microbiome is a dense and diverse ecosystem residing in the gastrointestinal tract and is primarily composed of bacteria, fungi, archaea, viruses, and protozoa.^1,2^ In recent decades, most research on the gastrointestinal microbiota has focused on commensal bacteria. Each gut contains 10^1 3^ bacteria, with approximately 100–500 different species per individual. Among these bacteria, the Bacteroidetes and Firmicutes phyla dominate the gastrointestinal tract, constituting 90% of the gut microbiome.^3,4^ However, interest in the “rare biosphere” of the gut,^5^ particularly fungi, is increasing. Although studies on gut fungal communities are in their infancy, there is increasing attention to this topic, as fungi may play a significant role in human health and disease.^6^ Fungi are commensal members of the host; they are prevalent in various body sites, such as the gut, lungs, and skin; and their composition varies significantly by niche.^7^ Compared with the variety and quantity of bacteria, fungi constitute only 0.01%–0.1% of the human gut microbiome.^8,9^ However, fungal cells are ten times wider and one hundred times larger in volume than bacterial cells are.^10^ Additionally, compared to bacteria, fungi possess larger genomes, greater genetic variability, greater morphological diversity, and unique metabolic and immunoregulatory functions. The healthy human gut harbors diverse fungal communities, mainly comprising Ascomycota, Basidiomycota, and other phyla.^9,11^ *Saccharomyces cerevisiae* and *Candida* species are particularly abundant in the gut. Gut fungi can confer health benefits or cause harm.

Under normal circumstances, the gut fungal community exhibits stability, dynamism, and symbiotic interactions with the host. However, factors such as diet, antimicrobials use, geography, and age can lead to gut fungal dysbiosis.^12^ The use of antimicrobials, particularly antibiotics, can cause gut fungal dysbiosis. For example, penicillin, clindamycin, and vancomycin promote *Candida albicans* overgrowth in the gut.^13^ Fungi are an integral part of the human gut microbiome and play a vital role in shaping host immunity and maintaining gut homeostasis. Under normal conditions, fungi and bacteria in the animal gut exist in a stable antagonistic, synergistic, or symbiotic relationship. *Candida albicans*, a commensal fungus in humans, has become a widely used model organism for studying host‒fungal interactions. Epithelial barrier damage or dysbiosis promotes its infection, while epithelial cells regulate its commensal state by recruiting immune cells and inducing IL-22 to produce secretory IgA (sIgA) and β-defensins.^14–16^

In this review, we examine the composition of methods for studying gut fungi, the gut mycobiome and its influencing factors (such as diet and age), its relationship with host health and disease, and the importance of fungal-bacterial-host immune interactions. We highlight the potential links between the fungal microbiome and common intestinal and hepatic diseases and discuss the previously underrecognized impact of gut mycobiome-based treatments on disease therapy. Our aim is to provide new insights and directions for ongoing fungal research.

## 2. Methods for studying gut fungi

To investigate the gut mycobiome and its interactions with the immune system, it is imperative to identify and quantify the fungal component of the microbiome. Early research relied on culturing fungi from various anatomical sites; however, this approach has yielded limited progress in comprehensively describing symbiotic fungal communities. Over the past decade, DNA-based methods have enabled culture-independent detection and identification of fungi. The rapid advancement of gene sequencing technologies has significantly broadened the scope of fungal species identification beyond traditional culturing methods. Several molecular methods are used to study gut fungi, each with its own advantages, limitations, and application scenarios (Table 1). Researchers employ DNA metabarcoding, which targets the fungal 18S rRNA gene or the internal transcribed spacer (ITS) regions of ribosomal DNA (rDNA), to study the gut mycobiome. The ITS region, located between the small subunit (18S) and large subunit (28S) rRNA genes, is divided into two subregions: ITS1 and ITS2. Both ITS1 and ITS2 are widely used as DNA barcode markers for fungal identification due to their high variability among different fungal species.^17^

**Table 1.** Comparison of different methods for studying gut fungi.

Method	Target molecule	Advantages	Limitations	Application scenarios	Reference
Amplicon sequencing (ITS1/ITS2)	DNA (ITS regions)	High taxonomic resolution at species level; widely used DNA barcode; cost-effective	PCR biases; limited to known sequences; cannot distinguish live/dead organisms	Profiling fungal diversity; identifying species composition in gut samples	^17–19^
Amplicon sequencing (18S rRNA)	DNA (18S rRNA gene)	Broad detection of fungi including basal lineages; useful when ITS regions are not available	Lower taxonomic resolution; may not distinguish closely related species	Initial surveys of fungal presence; detecting overall fungal abundance	^20^
Metagenomic shotgun sequencing	Total DNA	Comprehensive analysis of all genetic material; detects fungi, bacteria, viruses; potential to identify novel organisms	Higher cost; computationally intensive; fungal sequences may be underrepresented due to bacterial dominance	Exploring fungal gene content and potential functions; discovering new fungal species	^1,21,22^
Metaproteomics and metabolomics	Proteins/Metabolites	Direct measurement of functional activity; identification of microbial interactions and metabolic pathways	Technically challenging; lower throughput; difficulty in linking proteins/metabolites to specific organisms	Investigating functional interactions between fungi and host; identifying biomarkers of fungal activity	^23^

ITS, internal transcribed spacer.

The differences between ITS1 and ITS2 regions are significant and influence the detection and characterization of fungal communities. ITS1 is situated between the 18S rRNA and 5.8S rRNA genes, whereas ITS2 is located between the 5.8S rRNA and 28S rRNA genes.^24^ ITS1 generally exhibits greater length variability compared to ITS2, which can complicate sequence alignment and phylogenetic analyses due to insertions or deletions.^18^ These variations in length and location contribute to differences in taxonomic resolution between the two regions. ITS1 may provide better resolution for identifying certain fungal groups such as Basidiomycota, while ITS2 has been found to be more effective for Ascomycota.^19,25^ Some studies have reported that ITS2 sequences offer higher species-level resolution and better discrimination among closely related species.^26^ Primer design and amplification efficiency also differ between ITS1 and ITS2. Primers targeting ITS1 and ITS2 vary in their specificity and universality. ITS1 primers may have mismatches with some fungal groups, leading to biased amplification.^27^ In contrast, ITS2 primers are often considered more universal and may amplify a broader range of fungi with higher efficiency.^28^ However, primer bias can still occur, affecting the relative abundance and diversity estimates of fungal communities.^29^ Reference database availability is another important consideration. Both ITS1 and ITS2 regions have extensive entries in reference databases like UNITE and GenBank; however, discrepancies exist in the representation of certain taxa.^30^ ITS2 has been proposed as a universal fungal barcode due to the availability of more comprehensive and curated reference sequences.^31^ Given these differences, the choice between ITS1 and ITS2 depends on the specific objectives of the study and the fungal taxa of interest. For gut mycobiome studies aiming for comprehensive community profiling, some researchers advocate using both ITS regions in parallel to maximize taxonomic coverage and resolution.^32^ Additionally, careful primer selection and optimization of PCR conditions are essential to minimize amplification biases and improve the accuracy of fungal diversity assessments.

Emerging evidence based on sequencing analyses suggests a potential role of fungal colonization in pancreatic cancer development and progression. Studies have identified *Malassezia* as a key fungal taxon enriched in pancreatic ductal adenocarcinoma (PDAC) tissues, suggesting its involvement in creating an oncogenic microenvironment.^33^ Aykut et al. demonstrated that Malassezia species, enriched in the PDAC microenvironment, can activate the complement cascade via mannose-binding lectin, promoting tumor growth.^34^ However, these findings are not without controversy. One critical challenge in mycobiome research, particularly with low-biomass samples such as pancreatic tissues, is distinguishing genuine microbial signals from potential contaminants introduced during sample collection, DNA extraction, or library preparation. For example, it has been highlighted that proper use of negative controls and systematic efforts to identify and remove sequencing contaminants are crucial for interpreting microbiome data from low-biomass samples.^35^

Furthermore, although Aykut et al. observed fungal dysbiosis in the pancreas and gut of PDAC mouse models, subsequent analyses did not find similar differences in fungal communities in human pancreatic tissues or stool samples. These contrasting results suggest that there is currently insufficient evidence to support the hypothesis that pancreatic or gut mycobiota directly promote human pancreatic carcinogenesis. This highlights the importance of employing standardized methodologies for generating and analyzing microbiome sequencing data, particularly from low-biomass samples, to improve reproducibility and reliability across studies. Re-analysis of datasets has further demonstrated that methodological refinements, including advancements in sampling, DNA extraction, and sequencing protocols, can significantly alter previously reported conclusions. Nevertheless, our understanding of the genetic and functional variability of the human gut mycobiome remains limited. Major challenges arise from the absence of a comprehensive reference genome database for gut fungi, hindering the classification of most fungal amplicons and limiting comprehensive investigations of the entire fungal microbiome.^36–38^ Moreover, the limited reference genomes restrict the in-depth exploration of gene expression in gut fungal species via metatranscriptomics and metaproteomics.

Recently, Yan and colleagues reported the creation of the Cultivated Gut Fungi (CGF) catalog comprising 760 fungal genomes derived from the fecal samples of healthy volunteers.^39^ This catalog encompasses 48 families and 206 species, with 69 previously unrecognized fungal species that lack genomic information in existing databases. The CGF catalog has more than quadrupled the genomic resources for gut fungal species and doubled the genomic resources for fungal protein families. This research, through large-scale cultivation and sequencing of human gut fungi, established the most extensive reference genome catalog for cultivated human gut fungi to date and included metabolic function analysis. These findings provide crucial reference data for studies on gut fungal community structures and biological functions. However, a notable limitation of the current CGF catalog is the paucity of cultured fungi from sources outside China. While studies have reported the widespread distribution of CGF species in non-Chinese populations, specific gut fungi unique to these populations remain undiscovered.

## 3. Composition of and factors influencing the gut fungal community

### 3.1. Composition of the gut fungal community

Fungi are integral symbiotic members of the host; they are ubiquitously present in various body sites, such as the gut, lungs, and skin; and their composition significantly differs according to the ecological niche.^7^ As a crucial component of the gut microbiome, the collective composition, genes, and metabolites of symbiotic gut fungi are referred to as the “mycobiome.” Typically, up to 1,000 fungal cells can be detected per milliliter or gram of gut contents.^40^ The anatomical and physiological characteristics of the mouth, stomach, and intestines provide distinct ecological niches, leading to the formation of site-specific microbial communities. The total number of fungi increases progressively from the ileum to the colon, reaching the highest density in the distal gut of most monogastric animals.^41^ Healthy human bodies host a diverse array of fungi, with at least 66 genera and approximately 180 species described, along with several previously unknown fungi. The gut mycobiome comprises members of the phyla Ascomycota, Basidiomycota, and others. Additionally, genera such as *Saccharomyces*, *Candida*, *Malassezia*, *Cyberlindnera*, *Penicillium*, *Cladosporium*, and *Aspergillus* are commonly found in the human gut. Among these strains, *Saccharomyces cerevisiae* and *Candida* are particularly abundant.^42,43^

Gut fungi play dual roles, promoting and impairing health. *Candida albicans* is one of the most extensively studied fungi concerning human health. This fungus is normally detected in feces and is considered a normal constituent of the human gut microbiome. In immunocompetent individuals, *Candida albicans* is typically harmless and maintains a balanced relationship with other microorganisms.^44^ However, numerous studies have reported the pathogenic effects of *Candida albicans*. For example, in mouse models of DSS-induced colitis, mice colonized with *Candida albicans* exhibit more severe colitis than noncolonized mice do.^15,45,46^ Furthermore, *Candida albicans* can translocate from the gut to the bloodstream, potentially invading nearly all visceral organs and causing invasive, life-threatening infections. *Saccharomyces cerevisiae* is commonly found in many foods and frequently appears in the gastrointestinal tracts of humans and mice. However, it remains unclear whether *Saccharomyces cerevisiae* truly colonizes the gut or exists as a transient “pass-through” fungus.^47^ *Saccharomyces boulardii* is widely used as an effective probiotic for preventing and treating pathogenic infections and gut complications. Dietary supplementation with *Saccharomyces boulardii* can enhance both humoral and innate immunity, improve gut epithelium integrity, and restore the gut microbiome composition.^48^

### 3.2. Factors influencing gut fungi

**Figure 1.**
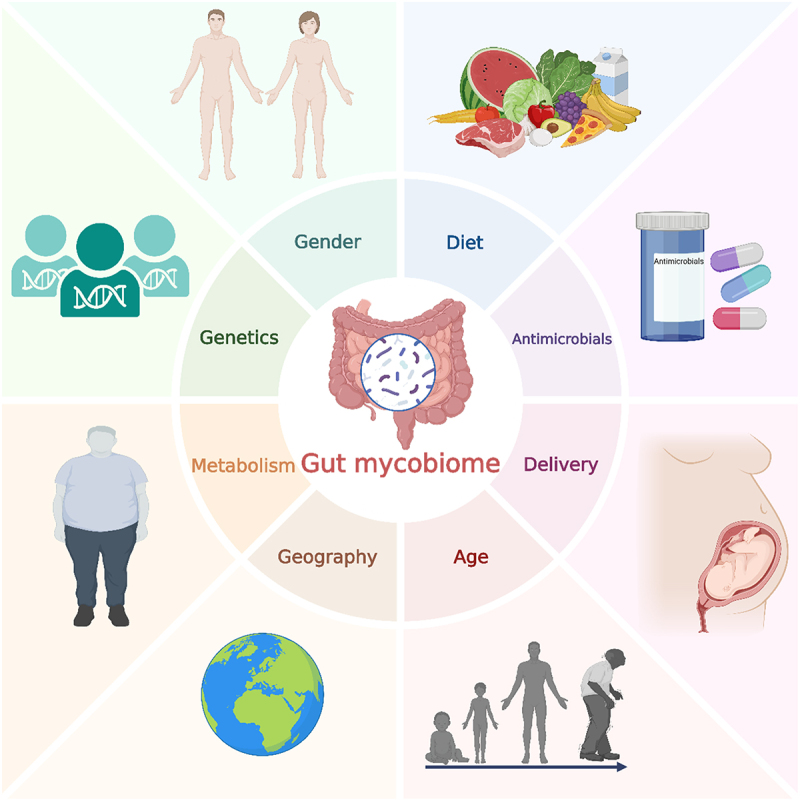
Factors influencing the gut mycobiome. This figure illustrates the primary factors influencing the composition and diversity of the gut mycobiome, ranked in order of importance starting from diet and proceeding clockwise: antimicrobials, delivery mode, age, geography, metabolism, genetics, and gender. These factors collectively shape the gut mycobiome and its impact on health and disease.

The composition and diversity of gut fungi are influenced by a myriad of factors, broadly categorized into age, mode of delivery, diet, host health, antimicrobials use, sex, and geographical environment (Figure 1). These factors collectively determine the diversity and composition of the gut mycobiome, subsequently impacting host health and disease status. Based on current evidence, diet appears to be the most significant factor shaping the gut mycobiome, followed by antimicrobials, particularly antibiotics and antifungals, and then mode of delivery and age. Factors such as host health status, geographical environment and sex differences seem to have a lesser impact. We evaluate and rank these factors according to their significance in influencing the gut mycobiome.

#### 3.2.1. Diet

Early research on gut mycobiomes focused on the impact of diet, as dietary intake and food choices are key factors causing individual differences in fungal colonization and composition.^49^ Diet plays a significant role in shaping the gut mycobiome, with specific dietary patterns influencing fungal diversity and composition. For instance, diets rich in fermented foods, bread, and beer are associated with a higher abundance of *Saccharomyces cerevisiae* in sequencing studies.^50–53^ However, a major limitation of sequencing-based mycobiome studies is the inability to differentiate between viable fungal cells and fungal DNA ingested with the diet.^54^ For example, diets containing foods produced with *S. cerevisiae* may lead to an increase in sequencing reads of *S. cerevisiae*, but it remains unclear to what extent this reflects dietary DNA rather than active colonization or proliferation of viable fungal cells.^55^ This limitation highlights the need for culture-dependent methods to verify findings and better interpret associations between dietary habits and gut fungal composition. In a study involving 98 healthy subjects, the abundance of *Candida* in fecal samples was positively correlated with the consumption of carbohydrate-rich diets and negatively correlated with the consumption of high-protein or high-fat diets.^56^ Another study revealed that compared with the consumption of plant-based diets, the consumption of diets rich in animal products increased the number of fungi in the human gut.^50^ The consumption of high-fat diets can alter the gut mycobiome composition, such as by reducing the *Saccharomyces cerevisiae* abundance, potentially leading to changes in the microbiome in mice.^57^ Further research on the interactions between dietary intake and fungal and bacterial species abundance revealed that stool samples from Indian and Japanese healthy subjects presented different fungal community structures.^58^ Compared with the Japanese population, the Indian population, whose diet is rich in plant polysaccharides, had higher abundances of *Prevotella* and *Candida* in their feces. The impact of diet on the gut mycobiome is multifaceted, with different dietary patterns and habits significantly altering the composition and diversity of gut fungi. Diet is the most significant factor shaping the gut mycobiome because it consistently influences fungal composition and diversity by modulating the gut environment, providing nutrients for specific fungi, and directly introducing fungal elements through food, making its effects pervasive and impactful across populations and conditions.

#### 3.2.2. Antimicrobials

Antimicrobials are among the common factors affecting gut fungal communities. The widespread use of antibiotics, particularly penicillins, clindamycin, and vancomycin, can deplete beneficial gut bacteria, creating conditions for fungal overgrowth, especially that of *Candida albicans*.^59^ While bacterial communities typically recover after antibiotic treatment, fungal communities may be affected long-term, leading to fungal overgrowth.^60^ Studies have shown that antibiotics that target anaerobes or broad-spectrum antibiotics can have varying effects on fungi, particularly *Candida albicans*.^61,62^ Additionally, patients with antibiotic-associated diarrhea often exhibit an overgrowth of *Candida* in their gastrointestinal tracts.^63^ Beyond antibiotics, other antimicrobials, including antifungal, antiviral, and antiparasitic agents, also influence the gut mycobiome. Antifungal medications, in particular, can profoundly alter fungal diversity and community composition. For instance, fluconazole treatment has been shown to significantly decrease the relative abundance of Ascomycota while increasing *Mucoromycota* in the intestines of treated mice compared to control groups, demonstrating its capacity to reshape fungal populations.^64^ Long-term use of antifungal agents can lead to the emergence of drug-resistant fungal strains or shifts in fungal species composition, with species such as *Candida glabrata* becoming more prevalent.^65^ However, fungi like *Candida albicans* can persist in the gut despite antifungal treatment due to biofilm formation and resistance mechanisms.^66^ While antiviral and antiparasitic medications are also widely used, studies focusing on their effects on the gut mycobiome are limited. Most research has concentrated on their impact on bacterial communities rather than fungi. Therefore, further studies are needed to elucidate how these agents alter the intestinal mycobiome. Given the profound impact of antibiotics and antifungals on gut fungal communities, medication use is ranked as the second most significant factor influencing the gut mycobiome.

#### 3.2.3. Mode of delivery and age

The presence of gut fungi is observable in early life, with fungal species and abundance changing as gestational age increases. Some studies suggest that fungal species aggregate in the gut before birth.^67^ However, this hypothesis remains controversial owing to significant debates over microbial colonization in utero versus postnatal gut fungal assembly.^68^ Like bacterial microbiomes, human mycobiomes are inherited at birth, during breastfeeding, and from close contacts.^69,70^ The mode of delivery significantly influences the composition of an infant’s gut mycobiome. Infants delivered vaginally are exposed to their mothers’ vaginal microbiota, resulting in increased gut fungal diversity, which aids in early immune system development and microbiome stability.^71^ In contrast, cesarean section (C-section)-delivered infants lack this exposure, leading to a gut mycobiome composition distinct from that of vaginally delivered infants, potentially increasing subsequent health risks. One study revealed that changes in the gut mycobiome composition during pregnancy are associated with prepregnancy weight status, with overweight or obese women exhibiting greater changes.^72^ Specifically, the presence of fungi of the genus *Mucor* in early pregnancy is positively correlated with the risk of gestational diabetes (GDM) and macrosomia, potentially independent of the impact of GDM on macrosomia. Furthermore, the abundances of specific genera such as *Aspergillus*, *Cladosporium*, *Penicillium*, and *Candida* were significantly lower in late pregnancy than in early pregnancy.

Research on neonatal mycobiomes indicates that the most abundant fungal species in the gut include *Candida parapsilosis*, *Candida tropicalis*, *Candida albicans*, *Saccharomyces cerevisiae*, and *Candida dubliniensis*, mirroring the mother’s vaginal microbiome.^73^ Initially, the infant gut is dominated by yeasts and *Malassezia* in the first month of life, with *Malassezia* abundance decreasing to undetectable levels by five months of age. This suggests that *Malassezia* may depend on factors present during early infancy, such as breastfeeding or formula feeding, and may disappear from the gut as these factors change. From three months to one year of age, the diversity and abundance of gut fungi increases, with *Saccharomyces cerevisiae* eventually replacing *Candida parapsilosis* and *Candida albicans* as the dominant fungi during this period. In healthy adult gut microbiomes, the genera *Candida*, *Saccharomyces*, and *Cladosporium* are the most prevalent.^74^ However, while *Saccharomyces cerevisiae* is often detected in adult guts, its presence largely depends on dietary intake of fermented foods and beverages.^50–53^ If these dietary components are not ingested, *S. cerevisiae* may be substantially reduced from the gut. In contrast, *Candida* species, such as *Candida albicans*, are able to persistently colonize the human gut irrespective of dietary habits or other micro environmental factors due to their adaptability and ability to utilize a variety of nutrients.^7^ Postnatally, gut fungal populations are influenced by various internal and external factors. Fungal diversity is lowest in adults but higher in infants and elderly individuals.^75^ Studies have reported higher proportions of *Aspergillus*, *Candida*, and *Davidiella* in elderly adults than in younger adults. The increased abundance of *Aspergillus* in the elderly may be attributed to environmental exposure (such as the inhalation or ingestion of spores) rather than persistent colonization, as *Aspergillus* species are commonly found in the environment and are unlikely to establish long-term residence in the gut.^76,77^ Conversely, compared with middle-aged individuals, elderly individuals exhibit reduced proportions of *Sporobolomyces* and *Agaricus* but increased proportions of *Malassezia*.^78,79^ Mode of delivery and age are thus important factors influencing the initial establishment and subsequent development of the gut mycobiome, ranked third in significance.

#### 3.2.4. Other factors

The geographical environment can lead to differences in gut fungal composition, but these differences may be less pronounced and often intertwined with dietary habits prevalent in different regions. For example, populations in industrialized areas predominantly harbor *Candida albicans*, whereas residents of remote rural areas are more likely to harbor *Candida krusei* and *Saccharomyces cerevisiae*.^80^ Sun *et al*.^81^ analyzed the fungal communities of rural and urban populations in China through metagenomic sequencing. Their results revealed that urban populations had a greater abundance of *Saccharomyces cerevisiae*, whereas the abundance of *Candida dubliniensis* was lower than that in rural populations. However, these variations may largely reflect differences in diet and lifestyle rather than the direct effect of geographical location.

The host’s health status can influence the gut mycobiome, but the associations are often confounded by diet. Obesity is associated with decreased gut fungal diversity, increased abundances of *Candida* and *Penicillium* and a decreased abundance of *Saccharomyces cerevisiae*.^82^ These associations, however, might partly result from dietary effects that influence both the mycobiome and host metabolism. For instance, diets rich in animal products may promote the growth of fungal taxa such as *Penicillium*, while plant-based diets are associated with *Candida* enrichment.^50^ This dual impact of diet complicates the interpretation of correlations between fungal taxa and metabolic disorders or obesity, as dietary habits may indirectly shape both fungal composition and host health.

Genetics also play a crucial role in the composition of the gut mycobiome. For example, single-nucleotide polymorphisms (SNPs) in the CARD9 gene are associated with the presence of *Malassezia* in the gut.^83^ These genetic factors may predispose individuals to colonization by specific fungal species, thus contributing to personalized mycobiome profiles. However, genetic factors seem to have a relatively minor influence compared to environmental factors such as diet and antimicrobials.

Sex differences may affect the diversity and composition of the gut mycobiome, but current evidence suggests that these effects are modest. For example, the genera *Aspergillus* and *Tremella* are more abundant in male subjects, whereas *Candida* is more prevalent in females, possibly because of the vaginal transmission of these fungi.^84^ Nevertheless, the overall impact of sex on gut mycobiome composition appears to be limited compared to other factors.

## 4. The complex interplay among gut fungi, gut bacteria, and host immunity

The interactions between gut microorganisms and the host immune system establish an ecological balance, enabling the host immune system to recognize invading pathogens to initiate immune responses while also achieving immune tolerance toward the commensal microbiota, thus maintaining gut microbiota homeostasis.^85^ From early training of the immune system during infancy to maturation in young individuals and a decline in middle-aged individuals, the close interactions among the bacterial microbiota, fungal microbiota, and gut mucosal immune system play a vital role throughout the host’s lifespan (Table 2).

**Table 2.** The role of common gut symbiotic fungi in host immunity.

Microorganism	Host	Key findings	Reference
*C. albicans*	Humans, mice	*C. albicans* is the major fungal inducer of the Th17 response, and intestinal inflammation can expand C. albicans-specific and cross-reactive Th17 cells	^86^
		*C. albicans* may mediate an enhanced Th2 response through ILC2 to exacerbate allergic airway inflammation	^87^
		Intestinal colonization by *C. albicans* reduces the antitumor immune response induced by radiation therapy	^88^
		*C. albicans* is the main inducer of antifungal immunoglobulin G (IgG), and the production of antifungal IgG depends on innate immune regulators CARD9 and CARD9 + CX3CR1 + macrophages	^89^
		*C. albicans*-specific Th17 cells migrate to the liver via Kupffer cells expressing IL-17 receptor A signaling, promoting liver disease	^90^
		*C. albicans* interacts with intestinal epithelial cells (IECs) via dectin-1, activating the Wnt signaling pathway to promote IEC proliferation, advancing CRC progression	^91^
*Malassezia*	Humans, mice	*Malassezia* primarily induces innate inflammation through CARD9 and is recognized by antifungal antibodies in Crohn’s disease patients	^83^
		*Malassezia* drives complement cascade activation via mannose-binding lectin, accelerating the onset of pancreatic cancer	^34^
*C. tropicalis*	Mice	*C. tropicalis* plays a decisive role in driving neo-mi DC migration	^92^
*C. parapsilosis*	Mice	*C. parapsilosis* produces fungal lipase, leading to an increase in free fatty acids in the gut, promoting obesity in mice	^93^
Mucosa-associated fungi	Humans, mice	Mucosa-associated fungi are related to protective immunity and epithelial barrier function, potentially driving neuroimmune regulation in mouse behavior through complementary type 17 immune mechanisms	^94^

Th17, T-helper 17 cells; Th2, T-helper 2 cells; ILC2, innate lymphoid cells type 2; IgG, immunoglobulin G; CARD9, caspase recruitment domain family member 9; CX3CR1, CX3C chemokine receptor 1; IL-17RA, interleukin-17 receptor A; Dectin-1, dendritic cell-associated C-type lectin-1; Wnt, Wingless-type mouse mammary tumor virus integration site signaling pathway; IEC, intestinal epithelial cell; CRC, colorectal cancer; DC, dendritic cells.

### 4.1. Interactions between gut fungi and host immunity

Fungi are common inhabitants of the gut barrier surface. In a healthy state, the relationship between commensal fungi and the host is mutualistic, with the gut mucosal immune system permitting the presence of fungi. Gut fungi play a role in immune protection. In the symbiotic state, *Candida albicans* induces CARD9+ CX3CR1+ macrophage-mediated production of antifungal immunoglobulin G (IgG), safeguarding the host from systemic attacks by fungal and bacterial pathogens, including *Candida albicans* itself.^89,95^ Moreover, colonization of the gut by *Candida albicans* elicits a Th17-mediated immune response.^86,96^ As such, many studies have considered *Candida albicans* the primary inducer of human Th17 immune responses.^86^ Colonization of the mouse gut by *Candida albicans* can induce systemic Th17 immune responses,^96^ as well as sustained proliferation of bone marrow progenitor cells dependent on IL-6 R signaling,^97^ thereby protecting the host from invasive *Staphylococcus aureus* infections by. However, antifungal drugs that selectively affect gut fungi can eliminate the beneficial effects of commensal fungi. In colitis models, antifungal agents lead to the expansion of the populations of neutrophils, monocytes, Th1 cells, and Th17 cells, whereas in pulmonary allergy models, these agents cause the enrichment of eosinophils, Th2 cells, and IgE-producing B cells. Furthermore, antifungal agents promote the expansion of the population of filamentous fungi, such as *Mucor*, *Aspergillus*, and *Alternaria*, which proliferate significantly during fungal dysbiosis, thereby exacerbating gut inflammation and allergic airway diseases.^98–101^

The immune system has evolved to tolerate fungi and respond to them during injury or infection. However, fungal dysbiosis frequently occurs,^102–104^ impacting mucus production, epithelial function, and gut immune defenses, thus compromising the gut barrier and increasing its permeability.^105,106^ Changes in gut barrier permeability allow opportunistic fungi or other components to enter the circulation. The carbohydrate components of fungal cell walls, such as β-glucans, α-1,3-glucans, mannans, and mannoproteins, are pathogen-associated molecular patterns (PAMPs).^107^ Innate immune cells are crucial for clearing fungi and initiating adaptive immune responses during fungal infections.^108^ Neutrophils, CCR2+ Ly6C+ monocytes, CX3CR1+ mononuclear phagocytes, and CD11b+ CD103+ dendritic cells constitute the first line of defense against fungal pathogens.^109^ These cells recognize key components of fungal cell walls through their pattern recognition receptors (PRRs) to drive antifungal immune responses. Macrophages and monocytes exhibit memory properties toward fungal cell wall components (e.g., β-glucans and chitin), protecting mice from secondary fungal invasion and enhancing host immunity against fungi. Additionally, gut fungi influence the formation and response of the host’s extra gastro intestinal immune system. Research suggests that commensal fungi may be involved in the maturation of secondary lymphoid organs, which are crucial sites for immune cell proliferation and immune responses, forming the body’s second line of immune defense.^92^ In early life, commensal fungi induce the migration of CD45+ CD103+ RALDH+ dendritic cells to surrounding lymph nodes. In germ-free (GF) mice, stimulation by *Candida albicans* in the gut triggers the migration of CD45+ CD103+ RALDH+ dendritic cells from the gut lamina propria to mesenteric lymph nodes, initiating lymph node structural development. These dendritic cells use retinoic acid-dependent signaling to induce lymphocyte homing to the gastrointestinal tract and surrounding lymph nodes. These findings indicate that innate and adaptive immune mechanisms mediated by gut fungi play indispensable roles in maintaining immune homeostasis.

### 4.2. Identification of common fungal patterns via pattern recognition receptors

**Figure 2.**
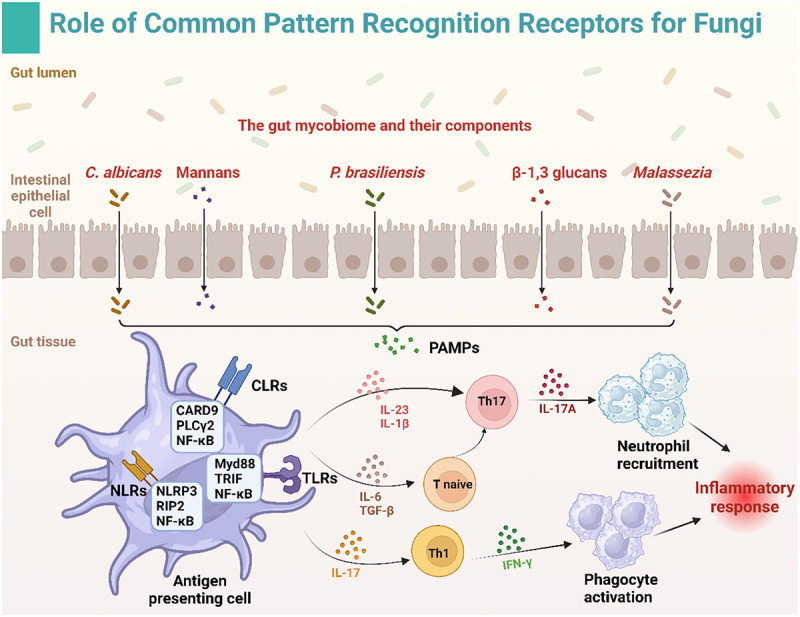
Roles of common pattern recognition receptors that detect fungal patterns. This figure illustrates the interaction between fungal components in the gut mycobiome and the host immune system. Key fungal components, including *Candida albicans*, mannans, *paracoccidioides brasiliensis*, β-1,3-glucans, and *Malassezia*, are recognized by pattern recognition receptors on antigen-presenting cells (APCs) in the gut. These receptors include C-type lectin receptors (CLRs), toll-like receptors (TLRs), and Nod-like receptors (NLRs), which trigger downstream signaling pathways involving CARD9, MyD88, and NF-κB. The activation of these pathways leads to the production of cytokines such as IL-23, IL-1β, IL-6, TGF-β, and IL-17, which promote Th17 and Th1 responses. These immune responses result in the recruitment of neutrophils, the activation of phagocytes, and the initiation of inflammatory responses to combat fungal infections.

PRRs are critical molecules for recognizing PAMPs, triggering signaling cascades in the immune system, activating the release of various cytokines, and inducing immune and inflammatory responses, thus influencing disease onset and progression. The main PRRs include C-type lectin receptors (CLRs), nucleotide-binding oligomerization domain (NOD)-like receptors (NLRs), and Toll-like receptors (TLRs). These receptors detect specific molecular structures of pathogens, such as fungi, to initiate host defense mechanisms, regulate immune responses, and maintain health and immunological balance (Figure 2).

#### 4.2.1. The role of CLRs in antifungal immunity

CLRs are crucial pattern recognition receptors that recognize carbohydrate molecules and play key roles in innate immunity, particularly in antifungal immunity. CLRs initiate immune responses by recognizing PAMPs, regulating host defense mechanisms against fungal infections. Major CLRs include dectin-1, dectin-2, dectin-3, macrophage inducible C-type lectin (MINCLE, also known as CLEC4E), and the mannose receptor, which recognize various molecules present in fungal cell walls.^110,111^ Genetic evidence and experimental studies suggest that CLRs play a central role in antifungal immunity, whereas TLRs and NLRs play secondary roles.^111^

Dectin-1, one of the most extensively studied CLRs, recognizes fungal β-glucans and plays a pivotal role in phagocytosis, fungal killing, and induction of cytokine production. Mouse model studies have revealed that dectin-1 provides protection against fungal infections in systemic candidiasis^112^ and in the skin,^108^ oral cavity,^113^ gut,^102^ and lungs.^114^ Human studies also indicate that mutations in the dectin-1 gene CLEC7A reduce dectin-1 expression, impairing cytokine production and predisposing individuals to recurrent vulvovaginal infections.^115^ The role of dectin-1 extends beyond local defense; during fungal dysbiosis, dectin-1 regulates antifungal immunity to prevent the overgrowth of opportunistic fungi such as *Candida* and *Mucor*. Correspondingly, dectin-1-deficient mice exhibit severe colitis and overgrowth of opportunistic fungi such as *Candida* and *Trichosporon* during gut inflammation.^102^

Dectin-2, encoded by CLEC6A, recognizes mannans in fungal cell walls, inducing Th17 responses that protect against systemic candidiasis.^116^ Studies suggest that dectin-1 and dectin-2 synergistically induce optimal Th17 responses against *Candida albicans*.^117^ However, the specific role of dectin-2 in mucosal immunity remains to be fully elucidated, with evidence highlighting its importance in skin and pulmonary fungal infections.^118,119^

Dectin-3 and MINCLE also play significant roles in antifungal immunity. Dectin-3 induces MINCLE expression and forms heterodimers with dectin-2 to increase signaling and immune responses.^120,121^ Mice lacking dectin-3 exhibit severe colitis, reduced Th17 cell numbers, and increased *Candida tropicalis* burdens.^122^ These findings suggest that dectin-3 maintains gut epithelial integrity and controls fungal burdens and inflammatory responses by regulating macrophage phagocytosis and Th17 cell numbers. MINCLE recognizes lipophilic components of *Malassezia*,^123^ and its specific functions in the gut and other mucosal sites warrant further research.

Spleen tyrosine kinase (SYK) is a key molecule in CLR signaling. The activation of CLRs leads to the phosphorylation of the ITAM motif by SRC family kinases, which recruit and phosphorylate SYK. This phosphorylation activates downstream signaling cascades, including activation of phospholipase Cγ2 (PLCγ2), protein kinase Cδ (PKCδ), and the CARD9–BCL–10–MALT1 complex.^14,110,111^ CARD9 is a crucial adaptor in CLR signaling pathways. CARD9-deficient mice exhibit significant changes in gut fungal and bacterial communities, including notably reduced *Lactobacillus* abundance, impacting ILC3 and Th cell IL-22 release.^124,125^ CARD9 deficiency also affects gut fungal immune regulation, influencing colorectal cancer development.^98,126,127^

#### 4.2.2. The role of CLRs in tumor immunity

The role of CLRs in tumor immunity is complex, with potential both for antitumor activity and for promoting tumor progression under specific conditions. For example, dectin-1 plays dual roles in hepatocellular carcinoma (HCC), enhancing NK cell cytotoxicity against tumors via N-glycan recognition in dendritic cells and macrophages.^128^ In some cancers, macrophage programming and adaptive immune suppression are promoted through interactions with galectin-9, thereby accelerating tumor progression.^129^ Dectin-2 expression in hepatic macrophages is crucial for inhibiting liver metastasis of colon cancer and melanoma.^130^ Conversely, dectin-3 deficiency impairs macrophage-mediated clearance of *Candida albicans*, increases the fecal fungal burden, and promotes the development of colitis-associated colorectal cancer.^131^ MINCLE plays protumorigenic roles in some cancers, for example, enhancing non-small cell lung cancer invasiveness and progression by promoting macrophage M2 polarization.^132^ Understanding the multifaceted roles of CLRs in antifungal immunity and cancer is essential. By recognizing fungal cell wall components, CLRs activate various signaling pathways, modulating host immune responses. While CLRs are central to antifungal defense and protect the host through diverse mechanisms, their roles in cancer warrant further investigation. Future research should elucidate the intricate mechanisms by which CLRs regulate immune responses and disease progression, providing a theoretical basis for the development of novel therapeutic strategies.

#### 4.2.3. The role of other antifungal immune receptors

In addition to CLRs, other molecules and mechanisms play crucial roles in antifungal immunity. Inflammasomes are important sentinels of the host’s innate immune defense against microbial infections. *Aspergillus fumigatus* galactosaminogalactan is a PAMP that activates the NLRP3 inflammasome.^133^ Inflammasomes such as NLRP3 and NLRC4 are key players in mucosal immunity during fungal infections. These inflammasomes activate caspase-1, which processes pro-IL-1β into its bioactive form, IL-1β, a critical mediator of fungal mucosal immunity.^14,113,134^ Studies have shown that the NLRP3 and NLRC4 inflammasomes protect mice from vaginal *Candida* infections by activating caspase-1 and IL-1β. Genetic polymorphisms in NLRP3 are associated with susceptibility to recurrent vulvovaginal candidiasis, increased IL-1β production, and excessive inflammation.^113,135^ Additionally, *Candida albicans* activates the NLRP3 inflammasome, inducing caspase-1 activation and IL-1β secretion, with NLRP3-deficient mice being more susceptible to *Candida* infections.^136^ Notably, NLRP3 activation appears to depend on fungal morphology; it is preferentially activated by *Candida* hyphae and hypha-secreted molecules, although the role of these molecules in controlling fungal populations during dysbiosis remains unclear.^14,137^

Despite advances in antifungal immunity research and the increasing availability of antifungal drugs, the rapid increase in the incidence of fungal infections necessitates new immunomodulatory strategies. Recent studies have indicated that inhibiting JUN N-terminal kinase 1 (JNK1) can promote the expression of CD23 (a newly identified CLR) and increase nitric oxide production, effectively combating fungal infections both in vitro and in vivo.^138^ PD-L1 (programmed death-ligand 1), long esteemed as a pivotal immune checkpoint molecule, has emerged as a cornerstone in the realm of oncological therapeutics. In a groundbreaking study, Kai Li et al. harnessed the cutting-edge technique of proximity labeling of phagosome proteomes (PhagoPL) to discern proteins that are distinctively enriched in phagosomes containing diverse microorganisms, including yeast and bacteria.^139^ The results unveiled a significant enrichment of PD-L1 within yeast-containing phagosomes. Further investigation illuminated that PD-L1 acts as a recognition receptor by binding to the fungal protein Rpl20b, thereby fostering the secretion of the anti-inflammatory IL-10 by macrophages. This interaction orchestrates the modulation of macrophage innate immune recognition responses to fungi. This pioneering discovery reveals for the first time the novel function of PD-L1 as a receptor for fungal binding.

TLRs also play significant roles in antifungal immunity. TLR2 is activated by *Candida albicans* phospholipomannan and *Aspergillus fumigatus* α-1,3-glucan, whereas TLR4 is activated by *Candida albicans* O-linked mannan and *Aspergillus* mannan.^140–143^ Genetic variations in TLRs are closely linked to fungal infection susceptibility, whereas these variations are often undetectable in healthy individuals. TLR1 variations increase the risk of candidemia,^144^ whereas polymorphisms in TLR1, TLR4, and TLR6 are associated with aspergillosis in allogeneic hematopoietic stem cell transplant recipients.^145^ TLRs can also activate signaling cascades via the TRIF and Myd88 pathways, leading to NF-κB nuclear translocation and the transcription of proinflammatory markers such as IL-6, IL-1β, and TNF-α. The release of these signaling molecules further induces Th1 and Th17 cell-mediated immune responses, which play a role in antifungal immunity.^146^

### 4.3. Interactions between gut fungi and bacteria

Gut fungi and bacteria share similar niches on the gut mucosal surface. These interactions can occur directly through physical contact and via secreted molecules or indirectly through alterations in host immune responses. The dynamic changes in interactions between gut fungi and bacteria underpin the mechanisms of disease pathogenesis and progression.^147^ The competitive interactions between gut fungi and bacteria have been the subject of extensive research. One study revealed that the commensal bacteria *Bacteroides thetaiotaomicron* and *Blautia producta* in mice can promote resistance to *Candida albicans* colonization by increasing the expression of the antimicrobial peptide LL-37, which is mediated by hypoxia-inducible factor 1α.^148^ A recent study revealed that *Lactobacillus rhamnosus* in the gut can reduce the pathogenicity of *Candida albicans* when it is metabolically active and proliferating.^149^ *Lactobacillus rhamnosus* colonization of gut epithelial cells results in the production of specific metabolites, such as phenyllactic acid, hydroxyphenyllactic acid, 2-hydroxyisocaproic acid, and 3-hydroxydecanoic acid, which antagonize *Candida albicans*. Furthermore, *Lactobacillus rhamnosus* depletes nutritional sources for *Candida albicans*, affecting its metabolism and transcription and forcing *Candida albicans* into an unfavorable growth environment. This results in altered energy metabolism and dysregulation of virulence-related genes, reducing the pathogenicity of *Candida albicans*. Thus, gut microbiota dysbiosis can lead to overgrowth of commensal *Candida*, which is a major trigger of infectious candidiasis. Gu et al. reported that *Candida albicans* levels were significantly reduced in patients with bacterial sepsis. Supernatants from *C. albicans* cultures were shown to markedly decrease bacterial loads and alleviate sepsis symptoms in mice subjected to cecal ligation and puncture and in pigs challenged with *Escherichia coli*. Further investigations revealed that phenylpyruvic acid, a metabolite derived from *C. albicans*, enhances macrophage bactericidal activity and reduces organ damage during sepsis.^150^

Commensalism is also a common example of bacterial‒fungal interactions. Clinical studies have revealed that *Bacteroides* in the human gut possesses glycoside phosphorylase genes that target β-1,2-mannosidic linkages in *Candida* mannan and can utilize yeast cell wall polysaccharides (such as mannans^151^ and β-glucans^152^) as a food source. Therefore, in addition to its acquisition of energy from dietary sources, gut *Bacteroides* may benefit from *Candida* colonization through this alternative mechanism. Similarly, *Candida albicans* supports the growth and proliferation of the commensal *Escherichia coli* K12 strain by providing siderophore-like molecules through an iron-responsive pathway.^153^ Moreover, studies have reported that *Candida albicans* supports the growth of strict anaerobes such as *Clostridioides difficile* under aerobic conditions.^154^ This phenomenon may involve mitochondrial redox mechanisms that result in oxygen consumption, creating a favorable environment for anaerobes.^155^ This significant finding in gut ecology explains why *Candida albicans* colonization markedly reduces the efficacy of fecal microbiota transplantation (FMT) in treating *Clostridioides difficile* infections (CDIs). These findings further indicate that *Candida albicans* colonization can inhibit the transplantation, colonization, and assembly of bacterial microbiomes in FMT recipients.^156^

Replacement is another phenomenon in bacterial – fungal interactions. Studies in mice have shown that after the depletion of symbiotic bacteria in the gut with antibiotics, mono colonization of *Candida albicans* or *Saccharomyces cerevisiae* in the mouse gut can actively calibrate the activation of protective CD8+ T cells.^157^ This effect can effectively reverse the susceptibility of mice to colitis and protect the host from influenza A virus infection. The protective effects of commensal fungi were later found to be mediated by mannans, which are highly conserved components of fungal cell walls. Stimulating the gut with this component alone can overcome disease susceptibility in bacterium-depleted mice. These results are analogous to the biological properties conferred by key molecular components of commensal bacteria. For example, the administration of lipoteichoic acid or LPS can prevent DSS-induced mortality in the absence of live commensal bacteria. Similarly, peptidoglycan, LPS, CpG, or poly(I:C) administration can enhance systemic antimicrobial immunity in antibiotic-treated mice. These findings underscore that commensal fungi can functionally replace gut bacteria by providing tonic microbial stimulation to protect local and systemic immunity.

## 5. The dysbiosis of gut fungi in intestinal and hepatic diseases

The proportions of Basidiomycota and Ascomycota within gut fungi remain relatively stable in healthy individuals. Gut fungi interact with intestinal epithelial cells and the immune system, contributing to the maintenance of gut barrier function and the regulation of both local and systemic immune responses. However, when the gut fungal community becomes dysbiotic, it can lead to the onset and progression of various diseases. Dysbiosis of gut fungi is considered a significant potential factor in IBD, Colorectal cancer (CRC), and various liver diseases (Table 3).

**Table 3.** Dysbiosis of gut fungi in intestinal and hepatic diseases.

Disease	Fungal changes	Relevant mechanisms	Reference
IBD (CD)	Increased abundance of *Candida albicans* and *Candida tropicalis*, decreased abundance of *Saccharomyces cerevisiae*	Colonization or infection with *Candida albicans* induces the production of anti-*Saccharomyces cerevisiae* antibodies (ASCAs)	^158^
IBD (CD)	Increased abundance of *Candida* spp., *Gibberella moniliformis*, *Alternaria brassicicola*, and *Cryptococcus neoformans*	Alterations in the composition of the intestinal fungal microbiota are associated with mucosal inflammation and exacerbated disease activity in CD	^159^
IBD (CD)	Increased abundance of *Candida*, *Clavispora*, *Cyberlindnera*, and *Kluyveromyces*	Fungal dysbiosis is the result of a combination of inflammation, antibiotic exposure, and dietary changes, with each factor exerting distinct influences on the fungal community composition	^160^
IBD (CD, UC)	Increased abundance of Basidiomycota and *Candida*, decreased abundance of Ascomycota and *Saccharomycetes*; *Malassezia* is enriched in UC but depleted in CD	Elevated fungal burden is observed during active intestinal inflammation in CD patients, while UC patients show a trend toward decreased intestinal fungal diversity	^161^
CRC	Increased abundance of *Malasseziomycetes* and *Aspergillus rambellii*, decreased abundance of *Saccharomycetes* and *Pneumocystidomycetes*	*Beauveria bassiana*-mediated upregulation of macrophage glycolytic pathways and IL-7 expression induces IL-22 secretion by type 3 innate lymphoid cells (ILC3s) through the AhR and STAT3 pathways, exacerbating CRC progression	^162^
CRC	Enrichment of *Candida albicans*	Intestinal epithelial cells may recognize *Candida albicans* via dectin-1, leading to the activation of the Wnt pathway and promoting CRC development	^91^
MASLD	Increased abundance of *Talaromyces*, *Paraphaeosphaeria*, *Lycoperdon*, *Curvularia*, *Phialemoniopsis*, *Paraboeremia*, *Sarcinomyces*, *Cladophialophora*, and *Sordaria*; decreased abundance of *Leptosphaeria*, *Pseudopithomyces*, and *Fusicolla*	MASLD patients exhibit a higher degree of fungal co-occurrence; several fungi are linked to liver damage, lipid metabolism, and MASLD progression	^163^
MASLD	Increased ratio of *Mucor* and *Saccharomyces*	The severity of MASLD correlates with specific compositions of fecal fungi; advanced fibrosis and severe MASLD are associated with elevated systemic anti-*Candida albicans* antibody levels	^164^
ALD	Increased abundance of *Candida*, decreased abundance of *Epicoccum*, unclassified fungi, *Galactomyces*, and *Debaryomyces*	Chronic alcohol consumption increases fungal translocation of β-glucans into the systemic circulation; antifungal treatment reduces gut fungal overgrowth and β-glucan translocation and ameliorates ethanol-induced liver disease in mice	^165^
ALD	Increased abundance of *Candida*, decreased abundance of *Penicillium* and *Saccharomyces*	Elevated serum ASCA levels are associated with increased mortality in alcoholic hepatitis patients	^166^
Liver cirrhosis	Significant decrease in abundance of Basidiomycota, increased abundance of Ascomycota	Ascomycota abundance is positively correlated with the incidence of end-stage liver disease and can be used to predict short-term hospitalization rates in advanced liver cirrhosis patients	^167^
Liver cirrhosis	Initial increase in abundance of *Chytridiomycota*, followed by a gradual replacement by Ascomycota during the progression from liver cirrhosis to hepatocellular carcinoma (HCC); dominance of *Kazachstania pintolopesii* in HCC group with significant reduction in abundance of *Saccharomyces cerevisiae*	Increased *Candida albicans* abundance and depletion of *Saccharomyces cerevisiae* may indicate the progression of liver cirrhosis to early-stage HCC	^168^
Liver cirrhosis	Increased relative abundance of *Aspergillus*, *Candida*, *Galactomyces*, *Saccharomyces*, and *Chaetomium*	Intestinal fungal diversity positively correlates with the progression of chronic HBV infection	^169^

IBD, inflammatory bowel disease; CD, Crohn’s disease; UC, ulcerative colitis; CRC, colorectal cancer; MASLD, metabolic dysfunction-associated steatotic liver disease; ALD, alcohol-associated liver disease; ASCAs, anti-Saccharomyces cerevisiae antibodies; IL-7, interleukin-7; IL-22, interleukin-22; ILC3s, type 3 innate lymphoid cells; AhR, aryl hydrocarbon receptor; STAT3, signal transducer and activator of transcription 3; HCC, hepatocellular carcinoma; HBV, hepatitis B virus.

### 5.1. Inflammatory bowel disease

IBD is a chronic inflammatory condition of the gastrointestinal tract that includes Crohn’s disease (CD) and ulcerative colitis (UC).^170^ UC is characterized by continuous, extensive, and superficial inflammation within the colon, whereas CD manifests as intermittent, penetrating lesions affecting various regions of the gastrointestinal tract. In IBD patients, changes in the structure and function of the gut microbiome, including bacteria, viruses, fungi, and protozoa, disrupt mucosal homeostasis, leading to persistent and excessive immune activation.^171^

Research has revealed significant dysbiosis in the fungal microbiota of IBD patients.^172^ Analysis of fecal samples from 235 IBD patients and 38 healthy controls via ITS2 fungal quantitative sequencing revealed an increased Basidiomycota/Ascomycota ratio at the phylum level.^173^ There was also an increase in *Candida albicans* abundance and a decrease in *Saccharomyces cerevisiae* abundance. Other studies have similarly reported elevated loads of *Candida albicans*, *Candida tropicalis*, and *Candida glabrata* in IBD patients.^161,174,175^ The relative abundance of *Candida albicans* in the feces of IBD patients is significantly associated with disease remission and relapse. For example, *Candida albicans* abundance markedly increases during IBD relapse compared with that during remission. *Candida tropicalis* interacts with anti-*Saccharomyces cerevisiae* antibodies (ASCAs), which are biomarkers known to be associated with CD^161,176^ that can be used to accurately identify individuals who will develop CD within five years.^177^ However, there is controversy regarding the proportion of *Saccharomyces* species. Sokol *et al*. reported a decrease in the relative abundance of *Saccharomyces* species,^161^ whereas Lewis *et al*. reported an increase in their abundance in CD.^103^ Methodological differences likely contributed to these discrepancies. Sokol et al. employed ITS2 sequencing, which provides detailed fungal profiles but is prone to biases in primer specificity and amplification, potentially affecting the detection of *S. cerevisiae*. In comparison, Lewis et al. used shotgun metagenomics, offering broader microbial coverage but often under representing fungal communities due to the dominance of bacterial DNA. Differences in the study populations further complicate interpretation, with Sokol et al.’s cohort comprising adult patients with diverse IBD phenotypes, while Lewis et al. focused on pediatric CD patients, whose microbiomes are influenced by developmental and dietary factors. Geographic and dietary variability may also play a role; Sokol et al.’s European cohort likely consumed diets rich in fermented foods, potentially influencing transient *S. cerevisiae* levels, whereas Lewis et al.’s North American cohort may have had different dietary exposures. Additionally, the studies differed in their focus on environmental influences, with Lewis et al. reporting a correlation between antibiotic exposure and increased fungal loads, including *S. cerevisiae*, a factor not addressed by Sokol et al. These differences in methodologies, patient demographics, and environmental factors underscore the critical need for standardized approaches to accurately characterize the gut mycobiome in IBD patients.

Most studies focus on characterizing the fungal microbiome in fecal or mucosal samples from CD patients or mixed IBD cohorts, with fewer studies specifically targeting UC patients. Some research has revealed compositional differences in the gut mycobiome between IBD patients and healthy individuals, yet there was no distinction between the fungal communities in UC and CD patients.^161,178^ Nonetheless, studies indicate elevated fungal loads during active intestinal inflammation in CD patients, whereas UC patients tend to exhibit reduced fungal diversity.^161^ This discrepancy could be due to CD often involving the terminal ileum, unlike UC. Pathophysiological changes in the terminal ileum might inhibit antimicrobial peptide production and bile acid reabsorption, promoting fungal growth. Consequently, CD patients have a greater abundance of *Candida* species than UC patients do. Li *et al*. reported significant alterations in the fungal composition of the inflamed mucosa, characterized by increased proportions of *Candida albicans*, *Gibberella*, *Trichosporon*, and *Cryptococcus neoformans*. The fungal richness and diversity in the inflamed mucosa are significantly greater than those in the noninflamed mucosa.^179^ These findings demonstrated that fungal microbiome diversity is associated with intestinal inflammation. Recent studies have also implicated *Malassezia* species in the pathogenesis of IBD. Limon et al. demonstrated that *Malassezia restricta* is enriched in the intestinal mucosa of CD patients compared to healthy controls.^83^ Moreover, gavage of *M. restricta* exacerbated colitis in mouse models of IBD. The exacerbation of colitis was dependent on the presence of the gene CARD9, which is associated with susceptibility to IBD in humans, indicating that host genetic factors can influence the impact of gut fungi on disease. Despite numerous studies indicating a correlation between gut fungal dysbiosis and IBD, the causal relationship remains to be determined. Therefore, large-scale, multicenter clinical studies are necessary to explore the role and mechanisms of the gut mycobiome in IBD.

### 5.2. Colorectal cancer

CRC has become the third most common cancer worldwide and the second leading cause of cancer-related death.^180^ One significant factor associated with CRC is the gut microbiome.^181,182^ The gut mycobiome is closely linked to the development and progression of colorectal cancer. However, only a few metagenomic studies have attempted to characterize the dysbiosis of the gut mycobiome in CRC, often with small sample sizes, making it challenging to draw definitive conclusions. Research by Coker *et al*. revealed that CRC patients present an increased Basidiomycota/Ascomycota ratio and a greater abundance of *Malassezia*, whereas the abundance of *Saccharomyces* and *Pneumocystis* decreases in these patients.^183,184^ The functional interaction of Dectin-3 with commensal fungi is crucial for maintaining colonic immune homeostasis. Studies have shown that the loss of the C-type lectin receptor Dectin-3 (Dectin-3−/−) leads to increased colorectal tumorigenesis and a greater burden of *Candida albicans* during chemically induced colitis.^185^ Elevated *Candida albicans* loads trigger macrophage glycolysis and IL-7 secretion, which, through the aryl hydrocarbon receptor and STAT3 pathways, induces RORγt innate lymphoid cells (ILC3s) to produce IL-22. Fungal load is positively correlated with IL-22 levels in the tumor tissues of CRC patients. Yu *et al*. utilized next-generation sequencing to analyze the ITS1 region of gut fungi in healthy individuals versus those with colorectal polyps or CRC. They reported that *Candida albicans* levels were significantly elevated in the guts of CRC patients. Further research indicated that the interaction of *Candida albicans* with dectin-1 in intestinal epithelial cells (IECs) activates the Wnt signaling pathway, promoting IEC proliferation, a pathway known to be involved in CRC progression.^91^ Wang *et al*. reported the presence of *Schizosaccharomyces pombe* in the fecal samples of both healthy individuals and CRC patients, with increased levels of proteins secreted from this yeast in CRC patients, including four proteins closely associated with CRC progression.^186^ A meta-analysis considered seven CRC microbiome studies, incorporating an additional internal cohort to reveal the distribution of fungal communities in CRC, totaling 1,329 metagenomes (from 454 CRC patients, 350 adenoma patients, and 525 healthy controls).^187^ This analysis revealed key CRC-associated fungi, including six enriched species (*Aspergillus rambellii*, *Cordyceps* sp. RAO-2017, *Erysiphe pulchra*, *Moniliophthora perniciosa*, *Sphaerulina musiva*, and *Phytophthora capsici*) and one depleted species (*Aspergillus kawachii*). Among these genera, *A. rambellii* was closely associated with the enrichment of *Fusobacterium nucleatum* in the gut of CRC patients. Functional validation experiments demonstrated that *Aspergillus rambellii* significantly promoted the growth of cancer cells in vitro and of tumors in vivo, confirming the potential causal relationship between fungal dysbiosis and CRC.

### 5.3. Hepatic diseases

The liver, the largest detoxification and metabolic organ in the human body, is intricately connected to the gut through the portal vein. This connection fosters a bidirectional relationship between the liver and the gut microbiota, significantly influencing metabolic, immune, and endocrine processes. This interaction is referred to as the “gut‒liver axis”.^188^ Dysbiosis in the gut mycobiome is closely linked to liver diseases, with changes in its composition potentially influencing the progression of chronic liver conditions.

#### 5.3.1. Metabolic dysfunction-associated steatotic liver disease

Metabolic dysfunction-associated steatotic liver disease (MASLD) is one of the most prevalent chronic liver conditions and is characterized by the accumulation of fat in the liver.^189,190^ Compared with patients with metabolic dysfunction-associated steatotic liver (MASL), those with metabolic dysfunction-associated steatohepatitis (MASH) face a greater risk of disease progression, which can lead to liver fibrosis, cirrhosis, and HCC.^191^ Currently, there is limited research on the relationship between gut fungi and MASLD. Demir and colleagues^164^ reported that the ratio of *Candida albicans/Saccharomyces cerevisiae* was significantly greater in nonobese MASH patients than in nonobese MASLD patients or control individuals. Additionally, in patients with MASH and F2‒F4 fibrosis, the *Mucor/Saccharomyces cerevisiae* ratio increased. In MASLD patients with type 2 diabetes, the *Malassezia/Saccharomyces cerevisiae* ratio was also significantly elevated. Another study demonstrated that supplementation with *Saccharomyces boulardii* could regulate the ratio of *Escherichia coli/Lactobacillus acidophilus* in the gut of MASLD model mice.^90^ Moreover, mice treated with *Saccharomyces boulardii* presented significant improvements in hepatic steatosis and inflammation. These findings suggest a potential link between gut bacteria and fungi in MASLD, although the specific mechanisms require further investigation.

#### 5.3.2. Alcohol-associated liver disease

Alcohol-associated liver disease (ALD) is one of the most common liver diseases worldwide and clinically manifests as alcohol-associated fatty liver, alcohol-associated hepatitis, alcoholic liver fibrosis, and alcoholic cirrhosis. The liver is the primary site for alcohol metabolism. When alcohol is consumed in excess over a prolonged period, it exceeds the liver’s metabolic capacity, leading to liver damage through various pathways and increasing the intestinal fungal load, resulting in changes in the abundance and composition of the gut microbiota.^90,192^ Studies have shown that dysbiosis of gut fungi plays a significant role in ALD development. Chronic alcohol consumption increases the number of gut fungi, facilitating the translocation of fungal β-glucans into the portal circulation. Further research indicated that β-glucan-induced liver inflammation primarily affects Kupffer cells via C-type lectin-like receptors (CLEC7A), increasing IL-1β expression, promoting hepatocyte injury, and promoting ethanol-induced liver disease. Additionally, antifungal treatment can reduce gut fungal growth and β-glucan translocation, thereby ameliorating alcohol-induced liver disease.^90^ A comparison between individuals with alcohol use disorder (AUD) and healthy controls revealed a marked increase in the abundance of *Candida*, *Debaryomyces*, *Pichia*, *Kluyveromyces*, *Issatchenkia*, *Candida albicans*, and *Candida zeylanoides* in AUD patients.^193^ Recent studies have also revealed an increase in the abundance of the gut fungus *Meyerozyma guilliermondii* in mice chronically fed ethanol, which, when cocultured with exogenous arachidonic acid, produces prostaglandin E2, promoting the development of alcohol-associated hepatitis syndrome (AAHS).^194^ These studies indicate that gut fungal dysbiosis may contribute to alcoholic liver disease progression. Changes in the fungal community may be associated with the effectiveness of abstinence interventions in patients with AUD. Compared with the control individuals, patients with AUD presented significant increases in the relative abundances of *Candida albicans*, *Debaryomyces*, *Pichia*, *Kluyveromyces*, and *Issatchenkia*. After abstinence intervention, patients whose liver function improved had a decreased relative abundance of *Candida albicans*, *Malassezia*, *Pichia*, *Kluyveromyces*, and *Issatchenkia* in the gut, as well as *Candida albicans* and *Candida tropicalis*, along with reduced levels of anti-*Candida albicans* IgG in the serum.^193^ Moreover, A study using a mouse model has further elucidated the role of fungi in ALD. Oral administration of *Malassezia restricta* has been shown to exacerbate ethanol-induced liver injury in both acute binge drinking and chronic ethanol feeding models.^195^ This effect is mediated by the C-type lectin receptor CLEC4N on Kupffer cells, which triggers an inflammatory cascade in response to *Malassezia*-derived antigens. These findings highlight a novel host-fungal interaction that contributes to the progression of ALD. Another study revealed that the *Candida albicans*-secreted exotoxin candidalysin damages hepatocytes in a dose-dependent manner in vitro and exacerbates alcohol-induced liver disease.^196^ This exotoxin, a pore-forming cytolytic peptide, is closely associated with the severity and mortality of alcohol-associated hepatitis. Candidalysin can induce a Th17 cell response^197^ and intracellular activation of the NLRP3 inflammasome in macrophages and dendritic cells,^137^ potentially amplifying liver inflammation. These findings suggest that gut fungal exotoxins, along with proinflammatory and anti-inflammatory factors, are involved in liver disease pathogenesis.

Moreover, an early study suggests that certain fungal signatures can differentiate between MASLD and ALD, which might have clinical implications in the future. Viebahn et al. compared the gut mycobiome of patients with ALD and MASLD and found distinct fungal profiles between the two groups.^198^ The study revealed that the genera, *Kluyveromyces*, and *Scopulariopsis*, along with species such as *Candida albicans* and *Malassezia restricta*, were significantly enriched in ALD patients. In contrast, the genera *Kazachstania* and *Mucor* were predominantly found in MASLD patients. These findings suggest that fungal signatures could serve as noninvasive biomarkers to differentiate ALD from MASLD, potentially aiding in diagnostic precision and influencing treatment strategies. However, further large-scale studies are necessary to validate these fungal signatures across diverse populations and clinical settings.

#### 5.3.3. Liver cirrhosis

Liver fibrosis and cirrhosis represent common pathological processes in chronic liver diseases. Advanced liver diseases such as alcohol-associated hepatitis, viral hepatitis, and fatty liver disease can lead to cirrhosis, which is characterized by the replacement of normal liver tissue with fibrotic and regenerative nodules, ultimately resulting in loss of liver function. When the disease progresses to the cirrhosis stage, gut fungi may become a primary factor contributing to overall microbial dysbiosis. Evidence suggests that the gut mycobiota may exhibit certain distinctions depending on the etiology of cirrhosis. Chen et al. reported that patients with HBV-related cirrhosis had higher levels of *Aspergillus*, *Candida*, *Galactomyces*, *Saccharomyces*, and *Chaetomium* compared to those with chronic HBV infection alone.^169^ Similarly, Yang et al. found that patients with alcohol-associated cirrhosis exhibited increased levels of *Candida* and *Pichia*, along with reduced gut fungal diversity, compared to healthy controls, MASLD, and ALD patients.^199^ Among alcohol-dependent individuals, *C. albicans* was identified as one of the most abundant *Candida* species in fecal samples; however, its relative abundance decreased with increasing liver disease severity, while *C. dubliniensis* became more prominent. Furthermore, serum samples from patients with alcohol-associated cirrhosis exhibited significantly higher levels of ASCA compared to those from controls or patients with HBV-related cirrhosis. Notably, ASCA levels were positively correlated with mortality in patients with alcohol-associated cirrhosis. In human cohorts, systemic exposure to fungal products was associated with mortality in alcohol-associated cirrhosis but not in viral hepatitis. These differences likely reflect variations in fungal exposure, alcohol-induced immunosuppression, and the specific metabolic or inflammatory environments associated with each etiology. However, as cirrhosis progresses to advanced stages, some studies indicate that the gut fungal microbiota converges into a similar dysbiotic state, characterized by the dominance of Ascomycota and a marked reduction in Basidiomycota.^167^ The abundance of Ascomycota has been positively correlated with the incidence of end-stage liver disease and can predict short-term hospitalization rates in advanced cirrhosis patients. In cirrhotic patients, irrespective of the underlying etiology, enrichment of *C. albicans* and depletion of other fungal taxa are consistently observed. This suggests that advanced liver dysfunction and associated changes, such as impaired gut barrier integrity, altered bile acid metabolism, and systemic inflammation, may homogenize the gut fungal microbiota across different etiologies.

A recent study using a DEN+CCl4-induced primary liver disease mouse model and clinical samples revealed longitudinal changes in gut fungi during primary liver disease progression.^168^ A previous study showed that as chronic liver disease progresses, the abundance of *Chytridiomycota* increases, and *Chytridiomycota* is then replaced by Ascomycota in HCC. Clinically, *Candida* (Ascomycota) and *Kazachstania pintolopesii* were predominant in the HCC group, whereas other fungi were depleted. The enrichment of *Candida albicans* and the depletion of *Saccharomyces cerevisiae* may indicate the progression of cirrhosis to early HCC. Supplementing *Candida albicans* and *Saccharomyces cerevisiae* in the diet during liver cirrhosis-HCC progression can either accelerate or delay HCC development. Thus, gut fungi may serve as biomarkers for liver disease progression and potential targets for preventive or therapeutic interventions. Currently, the relationship between chronic liver disease and fungi is poorly understood. Most studies have focused on the characteristics of the gut fungi in chronic liver disease patients, but the complex interactions between gut fungi and other microorganisms (such as bacteria and viruses) and how these interactions affect the host immune response remain unclear, and the specific mechanisms involved require further investigation.

## 6. Therapeutic strategies targeting gut fungi

**Figure 3.**
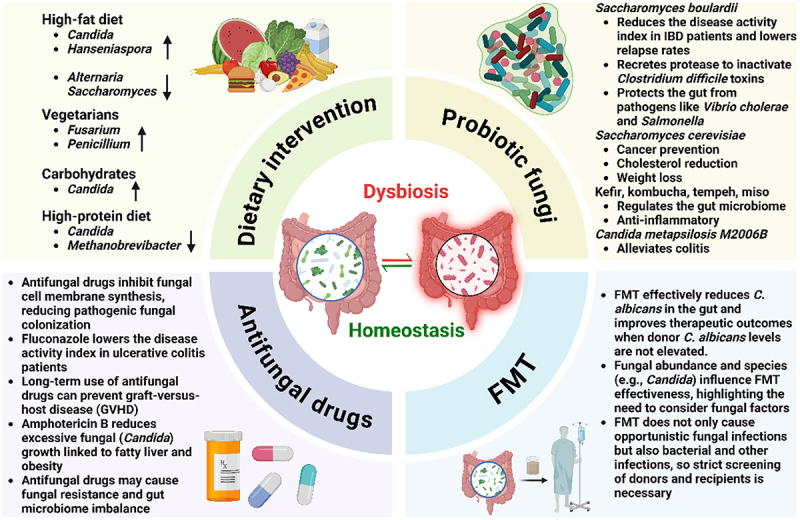
Therapeutic strategies targeting gut fungi, including dietary intervention, antifungal drugs, probiotic fungi, and fecal microbiota transplantation (FMT).

The gut microbiome plays a crucial role in maintaining human health, and its dysbiosis is closely linked to the onset and progression of various diseases. Modulating the gut microbiome, particularly the gut mycobiome, is considered a promising therapeutic approach. This section explores various strategies for modulating the gut microbiome, including dietary intervention, antifungal drugs, probiotic fungi, and FMT (Figure 3).

### 6.1. Dietary intervention

There is growing awareness of the significant influence of diet on the gut microbiota. According to reports, dietary changes can induce substantial and temporary microbial shifts within just 24 hours.^200^ Individuals with vastly different dietary patterns exhibit distinct gut microbiota compositions.^81^ Studies have shown that consumption of a high-fat diet in mice can lead to changes in fungal composition and reduced fungal diversity.^201^ For example, mice fed a high-fat diet exhibit increased levels of *Candida* and *Hanseniaspora*, whereas the abundance of *Alternaria* and *Saccharomyces* is lower than that in mice fed a standard diet.^202^ Vegetarians, on the other hand, display a gut fungal community with more spore-forming and dietary fungi (such as *Fusarium* and *Penicillium*) than meat eaters do.^202,203^ Additionally, research indicates that carbohydrates can alter the gut microbiota. An increase in dietary carbohydrate consumption leads to enrichment of *Candida* species, and consumption of diets rich in indigestible carbohydrates significantly increase the levels of *Bifidobacteria* and *Lactobacilli* in the gut.^204,205^ Conversely, consumption of high-protein diets is associated with a reduction in *Candida* and *Methanobrevibacter* abundance in healthy individuals.^56^ A study on the gut microbiota of Italian children revealed that increased intake of animal protein leads to increased levels of *Bacteroides* and *Alistipes*, whereas subjects consuming a protein-rich diet have fewer short-chain fatty acids in their stool.^206^ Mims *et al*.^207^ reported that the gut fungal community can be shaped by diet, with mice fed processed diets exhibiting different fungal compositions than those fed standardized diets, indicating that diet is a primary factor causing persistent differences in gut fungal communities.

Furthermore, fermented foods rich in various yeasts or filamentous fungi can serve as excellent sources of gut fungi and may offer benefits by modulating the gut microbiota composition and preventing diseases.^208^ For example, certain ethnic groups that frequently consume fermented foods, owing to their regional and cultural dietary habits, have a gut microbiota rich in *Saccharomyces*, which is correlated with lower incidences of IBD in these populations.^81^ Diets rich in coconut oil can reduce the availability of long-chain fatty acids in the gut, thereby decreasing the colonization of opportunistic pathogens such as *Candida albicans* in the gastrointestinal tract of mice.^209^ These findings underscore the close relationship between the diet and gut fungi. Dietary interventions represent a relatively innocuous and flexible approach to modulating the gut microbiome, including the fungal community, suggesting that dietary changes could be a promising strategy for treating various diseases.

### 6.2. Antifungal drugs

Antifungal drugs represent a traditional tool for directly targeting fungal infections. These drugs, such as fluconazole, amphotericin B, and caspofungin, act as tools to modulate the gut microbiota by inhibiting or killing pathogenic fungi, thereby restoring the microbial balance in the gut. By disrupting fungal cell membrane synthesis or function, these medications reduce fungal colonization in the gut. Clinically, antifungal drugs are often the first line of defense against fungal infections, preventing the growth and proliferation of pathogenic fungi. However, oral antifungal medications can significantly impact the gut fungal community structure, regardless of an individual’s health status. Fluconazole, a common antifungal, is used to treat fungal infections, such as candidiasis and candidemia, in immunocompromised IBD patients. In a study involving UC patients (*n* = 89), 20 patients with high gut fungal loads treated with fluconazole for four weeks presented a significant reduction in the disease activity index compared with those in the placebo and probiotic (Lacidofil) groups.^210^ Another randomized controlled clinical trial revealed that long-term fluconazole prophylaxis significantly reduced the incidence of severe gut graft-versus-host disease (GVHD) in allogeneic hematopoietic transplant recipients, highlighting the survival benefits of gut fungal community modulation in GVHD.^211^ However, prolonged fluconazole use can lead to gut microbial dysbiosis, exacerbating colitis and allergic airway diseases.^98^

Numerous studies have explored the potential of antifungal drugs to modulate gut fungal communities as a means for treating nonfungal infections. For example, fecal transplantation from MASH patients into GF mice, followed by dietary supplementation with amphotericin B, resulted in a significant reduction in the levels of fungi, including *Candida albicans*, *Mucor*, *Rhizopus*, and *Rhizomucor*, in the gut compared with those in control mice.^164^ Amphotericin B also lowered liver cholesterol and triglyceride levels and alleviated liver damage and steatosis in mice. Furthermore, *Candida glabrata* has been identified as a pathogenic fungus contributing to high-fat diet-induced obesity in mice.^212^ Oral administration of amphotericin B or fluconazole inhibited *Candida glabrata* proliferation in the gut, reducing the development of obesity and associated metabolic disorders in mice. These findings suggest that antifungal drugs can treat and prevent fatty liver disease and obesity by modulating the gut fungal composition or metabolites. Owing to cross-kingdom interactions between bacteria and fungi, oral antibiotics can also alter the structure of fungal communities.^213^ For example, penicillin and β-lactam antibiotics significantly reduce gut bacterial quantity and diversity, promoting the overgrowth of opportunistic fungi such as *Candida*.^88,213,214^

While antifungal drugs play a critical role in treating fungal infections and modulating the gut microbiome, long-term use may increase fungal resistance, complicating future treatment. These drugs can negatively impact the gut microbiome by disrupting beneficial microbial communities, weakening overall gut health. Therefore, the cautious use of antifungal drugs is essential, especially in patients with chronic inflammatory diseases, in which the gut microbiome is already imbalanced. The overuse of antifungal drugs may exacerbate this imbalance, adversely affecting patient health. Hence, treatment should balance the benefits and risks of these drugs, with consideration of adjunctive methods to support and restore gut microbiome balance.

### 6.3. Probiotic fungi

Probiotics are active microorganisms that confer health benefits to the host when they are administered in appropriate amounts.^215^ While bacterial probiotics such as *Lactobacillus* and *Bifidobacterium* are the most widely used, fungal probiotics and their derivatives also show potential in combating fungal infections and treating various diseases.^215,216^ Yeasts are among the most researched and promising fungal probiotics. *Saccharomyces boulardii*, which is closely related to *Saccharomyces cerevisiae*, is a probiotic strain known for its anti-inflammatory properties in colitis. Several studies have demonstrated that combining *Saccharomyces boulardii* with mesalazine significantly lowers the disease activity index score and reduces clinical relapse rates in IBD patients.^217^ *Saccharomyces boulardii* also lowers the risk of CDI, combats *Helicobacter pylori* infection, and alleviates symptoms associated with gastrointestinal infections.^215,218^ Therefore, *Saccharomyces boulardii* can be used as an adjunct therapy for IBD^219^ and irritable bowel syndrome (IBS).^220^ Its protective mechanism involves the secretion of proteases that can inactivate *Clostridium difficile* toxins A and B, thereby inhibiting *Clostridium difficile* proliferation.^221^ However, it remains to be fully determined whether this protective effect is entirely dependent on protease activity. Another potential protective mechanism against CDI is the stimulation of the production of IgA antibodies that target *Clostridium difficile* toxins.^222^ *Saccharomyces boulardii* also protects against some intestinal pathogenic bacteria, including *Vibrio cholerae*, *Salmonella typhimurium*, *Shigella dysenteriae*, and enterohemorrhagic *Escherichia coli* (EHEC).^223,224^ Both *Escherichia coli* and *Salmonella typhimurium* can bind to the surface of *Saccharomyces boulardii*, preventing their adhesion to intestinal epithelial cells and facilitating their rapid excretion via feces.^225,226^ *Saccharomyces boulardii* has been found to inhibit the colonization of *Candida albicans* and adherent-invasive *Escherichia coli* (AIEC), reducing colitis development in mice.

*Saccharomyces cerevisiae*, also known as brewer’s yeast, is another potential probiotic species. β-glucan extracted from the cell walls of *Saccharomyces cerevisiae* is a natural fungal product that may aid in cancer prevention and treatment, weight loss, cholesterol reduction, and enhanced radiation protection.^227,228^ Furthermore, purified glucose tolerance factor (GTF) from brewer’s yeast extract can lower plasma triglyceride, glucose, and cholesterol levels in genetically diabetic mice.^229^ Kefir is a probiotic beverage containing more than 50 species of yeasts and lactic acid bacteria.^230^ It has been shown to reduce the development of obesity and hepatic steatosis induced by consumption of a high-fat diet in mice by modulating the gut bacterial and fungal microbiota, promoting fatty acid oxidation, and reducing the levels of plasma inflammatory markers such as IL-6. In addition to kefir, other fermented foods rich in probiotic fungi include kombucha, tempeh, and miso. Kombucha is a fermented tea beverage containing a symbiotic culture of bacteria and yeasts (SCOBY), including species like *Zygosaccharomyces kombuchaensis* and *Brettanomyces bruxellensis*, which have been associated with antioxidant, antimicrobial, and detoxifying properties.^231,232^ Tempeh, a traditional Indonesian food made from fermented soybeans using the fungus *Rhizopus oligosporus*, is a rich source of protein. It has been shown to possess probiotic properties and offers potential health benefits for neurodegenerative diseases.^233^ Miso, a fermented soybean paste produced with the fungus *Aspergillus oryzae*, contains enzymes and bioactive compounds that can enhance digestion and exhibit anti-inflammatory effects.^234,235^

A recent study elucidated the biological effects and molecular mechanisms of the gut fungus *Candida metapsilosis* M2006B on colitis in mice.^236^ This study revealed that *Candida metapsilosis* M2006B significantly alleviates colitis in antibiotic-treated mice by activating the FXR. These studies indicate that *Saccharomyces boulardii*, *Saccharomyces cerevisiae*, and *Candida metapsilosis* M2006B have potential as beneficial gut fungi for the treatment and prevention of IBD and other metabolic diseases. Moreover, incorporating fermented foods rich in probiotic fungi into the diet may serve as a practical approach to regulating the gut fungal microbiome and promoting overall health. However, scientific data on the optimal dosage, safety, and therapeutic mechanisms of fungal probiotics are relatively limited, necessitating further research in these areas.

### 6.4. Fecal microbiota transplantation

FMT involves transferring fecal suspensions from healthy donors into the intestines of patients to restore the overall balance of the gut microbiota, thereby preventing the development of diseases associated with dysbiosis. FMT has shown an approximately 90% success rate in treating CDI.^237^ In 2013, the FDA approved FMT for treating recurrent CDI, making it the standard treatment for this condition.^238,239^ Although the efficacy of FMT has traditionally been attributed to bacterial influence, recent research suggests that the transfer of donor gut fungal communities during FMT may also impact treatment outcomes. Zuo and colleagues reported that gut fungal dysbiosis is associated with reduced efficacy of FMT in treating CDI.^240^ Their study revealed that donor gut fungi were significantly transferred to the recipient’s gut after FMT, and the success of FMT was related to an increase in the number of donor-derived fungal taxa in the recipient. The responders to FMT presented relatively high relative abundances of yeasts and *Aspergillus*, whereas the nonresponder and antibiotic-treated patients had fecal samples dominated by *Candida* species. CDI often involves enrichment of *C. albicans*, along with reduced fungal diversity, richness, and evenness. The responders to FMT treatment presented a decreased abundance of *C. albicans*, whereas the nonresponders maintained high levels of *C. albicans* in their feces. However, it should be clarified that FMT only reduces *C. albicans* if the donors do not have an increased relative abundance of *C. albicans*, highlighting the importance of donor selection. Similarly, in a mouse model, the presence of *C. albicans* negated the therapeutic effects of FMT on CDI. The efficacy of FMT was restored when antifungal agents were used to clear *C. albicans* in recipient mice.^241^ Another study on recurrent CDI reported that the abundance of *Yarrowia* species in the recipient’s gut before FMT was negatively correlated with the efficacy of FMT.^242^

In addition to treatment of CDI, FMT has also been reported for treating IBD. A randomized controlled trial involving UC patients (*n* = 24) described post-FMT fungal communities, highlighting the important association between the response and the *C. albicans* burden.^243^ A high pre-FMT abundance of *Candida* species in the gut was associated with an improved clinical response, whereas a reduced *Candida* burden post-FMT was correlated with improved disease severity. These findings suggest that FMT can reduce the *C. albicans* load, provided that the donor microbiota does not have a high abundance of *C. albicans*, and that the decrease in *C. albicans* abundance post-FMT contributes to positive therapeutic outcomes in both CDI and UC patients. Therefore, the effectiveness of FMT in reducing *C. albicans* depends significantly on the microbiota of the donors. The above findings suggest that FMT can reduce the *C. albicans* load and that the decrease in *C. albicans* abundance post-FMT contributes to positive therapeutic outcomes in both CDI and UC patients. These findings indicate a potential causal relationship between gut fungal dysbiosis and FMT outcomes, with *C. albicans* serving as a biomarker for predicting FMT efficacy. It is important to note that what is being transplanted via FMT depends significantly on the microbiota of the donors. The donor’s microbial composition, including bacteria, fungi, and viruses, can influence the efficacy and safety of FMT. Results of FMT for certain conditions can vary by geographical location and other factors, such as diet, lifestyle, and environmental exposures, which affect the donor microbiota.

To improve the likelihood of FMT success, proper preparation of the recipient is crucial. Pre-treatment with specific antibiotics can significantly reduce the existing gut bacterial load, creating a niche for the transplanted microbiota to colonize.^244^ However, antibiotics may also disrupt the fungal community, potentially leading to fungal overgrowth. Given the association of *Candida* species with reduced FMT efficacy, administering antifungal agents prior to FMT has been suggested to suppress pathogenic fungi like *C. albicans*, thereby enhancing FMT outcomes. In the mouse model described above, antifungal treatment with fluconazole before FMT improved the engraftment of donor microbiota and ameliorated disease symptoms.^241^ However, antifungal use must be carefully tailored to prevent excessive disruption of beneficial fungal taxa, which may also contribute to the therapeutic effects of FMT. Laxative-based bowel cleansing methods, such as polyethylene glycol (PEG), provide an effective and practical alternative to antibiotics for reducing gut microbial loads prior to FMT. PEG works by increasing fluid volume through osmotic flow, effectively flushing out luminal bacteria. Additionally, PEG introduces oxygen into the colon, a typically anaerobic environment, which diminishes nutrients available for resident anaerobic bacteria.^245^ Administering higher doses of PEG via oral gastric gavage has been shown to enhance bowel cleansing efficiency.^246^ Unlike antibiotics, PEG preserves indigenous microbiota post-treatment, avoiding the long-term microbiota disruption commonly associated with broad-spectrum antibiotics.^247^ However, side effects of PEG, including electrolyte imbalances and dehydration, must be carefully managed to minimize complications.^248^

The role of probiotics in pre-FMT preparation is still under investigation. Probiotics containing beneficial bacteria or fungi, such as *Saccharomyces boulardii*, have shown promise in reducing fungal overgrowth and supporting a balanced microbial environment. However, their specific impact on improving FMT efficacy remains unclear and requires further study. Dietary interventions aimed at modulating the gut microbiome may also be explored as adjunctive strategies. Additionally, emerging evidence suggests that the recipient’s immune status and gut inflammation levels may influence FMT outcomes. Immune-modulating agents or anti-inflammatory therapies prior to FMT may improve the engraftment of donor microbiota by reducing inflammation-induced barriers to colonization.^249^ Personalized approaches based on the recipient’s baseline microbiota composition, fungal burden, and immune status may further enhance FMT success rates. Thus, considering a combination of antibiotics, antifungals, PEG-based bowel cleansing, probiotics, and dietary interventions, along with personalized immune and microbiome assessments, may optimize the recipient’s gut environment and improve FMT outcomes.

FMT can cause not only opportunistic fungal infections but also bacterial and viral infections, especially if pathogens are present in the donor stool. Some patients, particularly those with compromised immune systems, may experience severe immune reactions following FMT. Therefore, it is essential that the potential adverse effects of FMT are cautiously considered. For example, FMT can potentially transfer various opportunistic fungi, such as *C. albicans*, *Candida glabrata*, *Candida tropicalis*, and *Candida dubliniensis*. Similarly, transmission of multidrug-resistant bacteria through FMT has been reported, leading to severe infections.^250^ Hence, it is crucial to minimize the risk of transmitting common pathogens by rigorously screening and managing the fungal components of both donors and recipients before conducting FMT to avoid severe fungal infections resulting from transplantation. Moreover, variations in donor microbiota due to geographical location, diet, and lifestyle factors can affect the composition of the transplanted microbiota and consequently the outcomes of FMT in different populations. Therefore, selecting appropriate donors and standardizing donor screening protocols are essential for maximizing the efficacy and safety of FMT across various settings.

## 7. Conclusion

Fungi, as crucial components of the human gut microbiome, significantly influence immune responses, health, and inflammatory disease development. Deep sequencing analyses have revealed significant differences in fungal communities across various populations, highlighting specific fungal species with potential relevance. These findings warrant further investigation. Gut fungi can modulate immune responses and potentially trigger intestinal inflammation, making them promising targets for microbiome therapies. FMT shows great therapeutic potential, but its wide range of effects necessitate the identification of specific fungi within the gut microbiome that cause or prevent diseases, along with their targets, to achieve precise regulation. There is still a long way to go to achieve these goals. The interactions between bacteria and fungi in the gut also need in-depth exploration. Studies have shown that bacteria influence fungal infections through various mechanisms. The intricate relationships among gut fungi, bacteria, and host immunity support the physiological homeostasis of the host, as well as the mechanisms of disease onset, progression, and treatment outcomes. The diverse roles of gut fungi are influenced by disease-specific and environmental factors, including the nonfungal microbiota, diet, urbanization, medication use, age, genetics, and immunity. The role of gut fungi in host health and disease is complex and critical to understand. While the full extent of the function of gut fungi within the microbiome remains incompletely understood, their potential as highly immunogenic components cannot be overlooked.

## 8. Outlook and future direction

Despite progress in understanding the role of the gut mycobiota in health and disease, much remains to be explored. Future research should focus on elucidating the causal relationships between gut fungal dysbiosis and disease pathogenesis. Longitudinal studies are needed to determine whether fungal community changes precede or follow disease onset, particularly in conditions such as IBD, MASLD, and ALD. The identification of fungal biomarkers for diagnostic and therapeutic purposes is another promising area of research. Fungal signatures, such as those distinguishing MASLD and ALD, could be further validated across diverse populations to develop noninvasive diagnostic tools. Similarly, exploring how fungal metabolites, such as phenylpyruvic acid from *C. albicans*, modulate immune responses and affect disease progression may offer novel therapeutic strategies. Additionally, the interplay between gut fungi, bacteria, and the host immune system requires deeper investigation. Understanding cross-kingdom interactions will provide insights into how fungal communities influence the efficacy of microbiome-based therapies and how fungal-bacterial synergies contribute to health or disease states. From a clinical perspective, personalized approaches to modulating the gut mycobiota through dietary interventions, probiotics, or antifungal therapies should be explored. These interventions could be tailored to an individual’s baseline microbiota composition, genetic predisposition, and immune status, offering new avenues for precision medicine. Finally, technological advancements in multi-omics, including fungal genomics, transcriptomics, and metabolomics, will be instrumental in uncovering the functional roles of gut fungi. Developing standardized protocols for fungal sample collection, processing, and analysis will ensure reproducibility and comparability across studies, accelerating progress in this emerging field. By addressing these gaps, future research has the potential to unlock the therapeutic potential of the gut mycobiota, transforming our understanding of its role in maintaining health and treating disease.

## References

[1] Qin J, Li R, Raes J, Arumugam M, Burgdorf KS, Manichanh C, Nielsen T, Pons N, Levenez F. Yamada TJn: a human gut microbial gene catalogue established by metagenomic sequencing. Nature. 2010;464(7285):59–35. 20203603 10.1038/nature08821 PMC3779803

[2] Suhr MJ, Banjara N. Hallen‐Adams HEJLiam: Sequence‐based methods for detecting and evaluating the human gut mycobiome. Abbreviation Title Lett. Appl. Microbiol. 2016;62(3):209–215. 10.1111/lam.12539 26669281

[3] Eckburg PB, Bik EM, Bernstein CN, Purdom E, Dethlefsen L, Sargent M, Gill SR, Nelson KE, Relman DA. Relman DAJs: diversity of the human intestinal microbial flora. Science. 2005;308(5728):1635–1638. doi:10.1126/science.1110591. 15831718 PMC1395357

[4] Arumugam M, Raes J, Pelletier E, Le Paslier D, Yamada T, Mende DR, Fernandes GR, Tap J, Bruls T, Batto J-M, et al. Batto J-MJn: enterotypes of the human gut microbiome. Nature. 2011;473(7346):174–180. doi:10.1038/nature09944. 21508958 PMC3728647

[5] Sogin ML, Morrison HG, Huber JA, Welch DM, Huse SM, Neal PR, Arrieta JM, Herndl GJ. Herndl GJJPotNAoS: microbial diversity in the deep sea and the underexplored “rare biosphere”. Proc Natl Acad Sci. 2006;103(32):12115–12120. doi:10.1073/pnas.0605127103. 16880384 PMC1524930

[6] Cui L, Morris A, Ghedin E. Ghedin EJGm: the human mycobiome in health and disease. Genome Med. 2013;5(7):1–12. doi:10.1186/gm467. 23899327 PMC3978422

[7] Underhill DM, Iliev ID. The mycobiota: interactions between commensal fungi and the host immune system. Nat Rev Immunol. 2014;14(6):405–416. doi:10.1038/nri3684. 24854590 PMC4332855

[8] Hmjrs C, Nelson KE, Weinstock GM, Highlander SK, Worley KC, Creasy HH, Wortman JR, Rusch DB, Mitreva M, Sodergren EJS. A catalog of reference genomes from the human microbiome. Science. 2010;328(5981):994–999. 20489017 10.1126/science.1183605 PMC2940224

[9] Nash A, Auchtung T, Wong M, Smith D, Gesell J, Ross M, Stewart C, Metcalf G, Muzny D, Gibbs R, et al. The gut mycobiome of the human microbiome project healthy cohort. Microbiome. 2017;5(1):153. In.; doi: 10.1186/s40168-017-0373-4. 29178920 PMC5702186

[10] Richard ML, Hjnrg S. Hepatology: the gut mycobiota: insights into analysis, environmental interactions and role in gastrointestinal diseases. Nature reviews. Gastroenterology & hepatology. 2019;16(6):331–345. 30824884 10.1038/s41575-019-0121-2

[11] Hallen-Adams HE, Suhr MJ. Fungi in the healthy human gastrointestinal tract. Virulence. 2017;8(3):352–358. doi:10.1080/21505594.2016.1247140. 27736307 PMC5411236

[12] de Oliveira GLV. Chapter 33 - the gut microbiome in autoimmune diseases. In: Faintuch J, and Faintuch S, editors. Microbiome and Metabolome in diagnosis, therapy, and other strategic applications. ed. Academic Press; 2019. p. 325–332.

[13] Kennedy MJ, PajsjoM V. Mycology V: effect of various antibiotics on gastrointestinal colonization and dissemination by Candida albicans. Med Mycology. 1985;23(4):265–273. doi:10.1080/00362178585380391. 3901329

[14] Netea MG, Joosten LA, van der Meer Jw, Kullberg BJ, van de Veerdonk Fl, van der Meer JWM, van de Veerdonk FL. Immune defence against Candida fungal infections. Nat Rev Immunol. 2015;15(10):630–642. doi:10.1038/nri3897. 26388329

[15] Ost KS, Tr O, Stephens WZ, Chiaro T, Zhou H, Penman J, Bell R, Catanzaro JR, Song D, Singh SJN. Adaptive immunity induces mutualism between commensal eukaryotes. Nature. 2021;596(7870):114–118. 34262174 10.1038/s41586-021-03722-w PMC8904204

[16] Doron I, Mesko M, Li K XV, Leonardi T, Shaw I, Fiers DG, Lin WD, Wy B-D, M RE, Román E, et al. Mycobiota-induced IgA antibodies regulate fungal commensalism in the gut and are dysregulated in Crohn’s disease. Nat Microbiol. 2021;6(12):1493–1504. doi:10.1038/s41564-021-00983-z. 34811531 PMC8622360

[17] Schoch CL, Seifert KA, Huhndorf S, Robert V, Spouge JL, Levesque CA, Chen W, Consortium FB, List FBCA. Bolchacova EJPotnaoS: nuclear ribosomal internal transcribed spacer (ITS) region as a universal DNA barcode marker for fungi. Proc Natl Acad Sci USA. 2012;109(16):6241–6246. doi:10.1073/pnas.1117018109. 22454494 PMC3341068

[18] Nilsson RH, Ryberg M, Abarenkov K, Sjökvist E, Ejfml K. The ITS region as a target for characterization of fungal communities using emerging sequencing technologies. FEMS Microbiol Lett. 2009;296(1):97–101. doi:10.1111/j.1574-6968.2009.01618.x. 19459974

[19] Bellemain E, Carlsen T, Brochmann C, Coissac E, Taberlet P, HJBm K. ITS as an environmental DNA barcode for fungi: an in silico approach reveals potential PCR biases. BMC Microbiol. 2010;10(1):1–9. doi:10.1186/1471-2180-10-189. 20618939 PMC2909996

[20] Hibbett DS, Binder M, Bischoff JF, Blackwell M, Cannon PF, Eriksson OE, Huhndorf S, James T, Kirk PM, Lücking R, et al. Lücking RJMr: a higher-level phylogenetic classification of the fungi. Mycol Res. 2007;111(5):509–547. doi:10.1016/j.mycres.2007.03.004. 17572334

[21] Huseyin CE, Rubio RC, O’Sullivan O, Cotter PD, Scanlan PD. Scanlan PDJFiM: the fungal frontier: a comparative analysis of methods used in the study of the human gut mycobiome. Front Microbiol. 2017;8:1432. doi:10.3389/fmicb.2017.01432. 28824566 PMC5534473

[22] Thomas T, Gilbert J, Meyer F. Meyer FJMi, experimentation: metagenomics-a guide from sampling to data analysis. Microb Inf Experimentation. 2012;2(1):1–12. doi:10.1186/2042-5783-2-3. PMC3351745 22587947

[23] Verberkmoes NC, Russell AL, Shah M, Godzik A, Rosenquist M, Halfvarson J, Lefsrud MG, Apajalahti J, Tysk C, Hettich RL, et al. Shotgun metaproteomics of the human distal gut microbiota. The ISME J. 2009;3(2):179–189. doi:10.1038/ismej.2008.108. 18971961

[24] White, TJ. PPAgtm, applications/Academic press I: amplification and direct sequencing of fungal ribosomal RNA genes for phylogenetics. PCR Protocols, a Guide to Methods and Applications. 1990;315–322.

[25] Blaalid R, Kumar S, Nilsson RH, Abarenkov K, Kirk P, HJMer K. ITS 1 versus ITS 2 as DNA metabarcodes for fungi. Mol Ecol Resour. 2013;13(2):218–224. doi:10.1111/1755-0998.12065. 23350562

[26] Wang XC, Liu C, Huang L, Bengtsson‐Palme J, Chen H, Zhang JH, Cai D, JQJMer L. ITS 1: a DNA barcode better than ITS 2 in eukaryotes? Mol Ecol Resour. 2015;15(3):573–586. doi:10.1111/1755-0998.12325. 25187125

[27] Ihrmark K, Bödeker IT, Cruz-Martinez K, Friberg H, Kubartova A, Schenck J, Strid Y, Stenlid J, Brandström-Durling M, KEJFme C, et al. New primers to amplify the fungal ITS2 region–evaluation by 454-sequencing of artificial and natural communities. FEMS Microbiol Ecol. 2012;82(3):666–677. doi:10.1111/j.1574-6941.2012.01437.x. 22738186

[28] Toju H, Tanabe AS, Yamamoto S, HJPo S. High-coverage ITS primers for the DNA-based identification of ascomycetes and basidiomycetes in environmental samples. PLOS ONE. 2012;7(7):e40863. doi:10.1371/journal.pone.0040863. 22808280 PMC3395698

[29] Smith DP, Peay KG. Peay KGJPo: sequence depth, not PCR replication, improves ecological inference from next generation DNA sequencing. PLOS ONE. 2014;9(2):e90234. doi:10.1371/journal.pone.0090234. 24587293 PMC3938664

[30] Abarenkov K, Nilsson RH, Larsson K-H, Alexander IJ, Eberhardt U, Erland S, Høiland K, Kjøller R, Larsson E, Tjtnp P, et al. The UNITE database for molecular identification of fungi–recent updates and future perspectives. New Phytol. 2010;186(2):281–285. doi:10.1111/j.1469-8137.2009.03160.x. 20409185

[31] Nilsson RH, Tedersoo L, Ryberg M, Kristiansson E, Hartmann M, Unterseher M, Porter TM, Bengtsson-Palme J, Walker DM, De Sousa FJM, et al. A comprehensive, automatically updated fungal ITS sequence dataset for reference-based chimera control in environmental sequencing efforts. Microbes Environ. 2015;30(2):145–150. doi:10.1264/jsme2.ME14121. 25786896 PMC4462924

[32] Lindahl BD, Nilsson RH, Tedersoo L, Abarenkov K, Carlsen T, Kjøller R, Kõljalg U, Pennanen T, Rosendahl S, JJNp S, et al. Fungal community analysis by high-throughput sequencing of amplified markers – a user’s guide. New Phytol. 2013;199(1):288–299. doi:10.1111/nph.12243. 23534863 PMC3712477

[33] Alam A, Levanduski E, Denz P, Villavicencio HS, Bhatta M, Alhorebi L, Zhang Y, Gomez EC, Morreale B, Senchanthisai S, et al. Fungal mycobiome drives IL-33 secretion and type 2 immunity in pancrea tic cancer. Cancer Cell. 2022;40(2):153–167.e111. doi:10.1016/j.ccell.2022.01.003. 35120601 PMC8847236

[34] Aykut B, Pushalkar S, Chen R, Li Q, Abengozar R, Kim JI, Shadaloey SA, Wu D, Preiss P, Verma N, et al. The fungal mycobiome promotes pancreatic oncogenesis via activation of MBL. Nature. 2019;574(7777):264–267. doi:10.1038/s41586-019-1608-2. 31578522 PMC6858566

[35] Fletcher AA, Kelly MS, Eckhoff AM, Allen PJ. Revisiting the intrinsic mycobiome in pancreatic cancer. Nature. 2023;620(7972):E1–e6. doi:10.1038/s41586-023-06292-1. 37532819 PMC11062486

[36] Suhr MJ, Banjara N, Hallen-Adams HE. Sequence-based methods for detecting and evaluating the human gut myco biome. Lett Appl Microbiol. 2016;62(3):209–215. doi:10.1111/lam.12539. 26669281

[37] Nash AK, Auchtung TA, Wong MC, Smith DP, Gesell JR, Ross MC, Stewart CJ, Metcalf GA, Muzny DM, Gibbs RA, et al. The gut mycobiome of the human microbiome project healthy cohort. Microbiome. 2017;5(1). doi:10.1186/s40168-017-0373-4. PMC5702186 29178920

[38] Huseyin CE, Rubio RC, O’Sullivan O, Cotter PD, Scanlan PD. The fungal frontier: a comparative analysis of methods used in the Study of the human gut mycobiome. Frontiers in Microbiology. 8;1432. 10.3389/fmicb.2017.01432 PMC5534473 28824566

[39] Yan Q, Li S, Yan Q, Huo X, Wang C, Wang X, Sun Y, Zhao W, Yu Z, Zhang Y, et al. A genomic compendium of cultivated human gut fungi characterizes the gut mycobiome and its relevance to common diseases. Cell. 2024;187(12):2969–2989.e2924. doi:10.1016/j.cell.2024.04.043. 38776919

[40] Schulze J, Sonnenborn U. Yeasts in the gut: from commensals to infectious agents. Deutsches Arzteblatt Int. 2009;106(51–52):837–842. 10.3238/arztebl.2009.0837 PMC2803610 20062581

[41] Scupham Alexandra J, Presley Laura L, Wei B, Bent E, Griffith N, M M, Zhu F, Oluwadara O, Rao N, Braun J, et al. Abundant and diverse fungal microbiota in the murine intestine. Appl Environ Microbiol. 2006;72(1):793–801. doi:10.1128/AEM.72.1.793-801.2006. 16391120 PMC1352209

[42] Raimondi S, Amaretti A, Gozzoli C, Simone M, Righini L, Candeliere F, Brun P, Ardizzoni A, Colombari B, Paulone S, et al. Longitudinal survey of fungi in the human gut: ITS profiling, phenotyping, and colonization. Frontiers in Microbiology. 2019;10:1575. 10.3389/fmicb.2019.01575 PMC6636193 31354669

[43] Zhang L, Zhan H, Xu W, Yan S, Ng SC. The role of gut mycobiome in health and diseases. Ther Adv Gastroenterol. 2021;14:17562848211047130. doi:10.1177/17562848211047130. PMC8474302 34589139

[44] Odds, FC. JCCRiM: Candida infections: an overview. Critical Reviews in Microbiology. 1987;15(1):1–5. 10.3109/10408418709104444 3319417

[45] Naglik JR, Gaffen SL. Hube BJCoim: Candidalysin: discovery and function in Candida albicans infections. Current Opinion in Microbiology. 2019;52:100–109. 31288097 10.1016/j.mib.2019.06.002 PMC6687503

[46] Leonardi I, Gao IH, Lin W-Y, Allen M, Li XV, Fiers WD, De CM, Putzel GG, Yantiss RK, Mjc J, et al. Mucosal fungi promote gut barrier function and social behavior via type 17 immunity. Cell. 2022;185(5):831–846. e814. doi:10.1016/j.cell.2022.01.017. 35176228 PMC8897247

[47] Auchtung TA, Fofanova TY, Stewart CJ, Nash AK, Wong MC, Gesell JR, Auchtung JM, Ajami NJ, Petrosino JF, Mitchell AP. Investigating colonization of the healthy adult gastrointestinal tract by Fungi. mSphere. 2018;3(2). doi:10.1128/mSphere.00092-18. PMC5874442 29600282

[48] Sen S, Tjjfg M. Biology: yeasts as probiotics: mechanisms, outcomes, and future potential. Fungal Genetics and Biology. 2020;137:103333. 31923554 10.1016/j.fgb.2020.103333

[49] Finegold SM, Attebery HR, Sutter VL. Effect of diet on human fecal flora: comparison of Japanese and American diets. The Am J Clin Nutr. 1974;27(12):1456–1469. doi:10.1093/ajcn/27.12.1456. 4432829

[50] David LA, Maurice CF, Carmody RN, Gootenberg DB, Button JE, Wolfe BE, Ling AV, Devlin AS, Varma Y, Fischbach MA, et al. Diet rapidly and reproducibly alters the human gut microbiome. Nature. 2014;505(7484):559–563. doi:10.1038/nature12820. 24336217 PMC3957428

[51] Fröhlich‐Wyder MT, Arias‐Roth E, Jakob E. Cheese yeasts. Yeast. 2019;36(3):129–141. doi:10.1002/yea.3368. 30512214

[52] Heitmann M, Zannini E, Arendt E. Impact ofSaccharomyces cerevisiae metabolites produced during fe rmentation on bread quality parameters: a review. Crit Rev Food Sci Nutr. 2018;58(7):1152–1164. doi:10.1080/10408398.2016.1244153. 27874287

[53] Alba-Lois L, Segal-Kischinevzky C. Yeast fermentation and the making of beer and wine. Nature education. Nature Education. 2010;3:17.

[54] Santus W, Rana AP, Devlin JR, Kiernan KA, Jacob CC, Tjokrosurjo J, Underhill DM, Behnsen J. Mycobiota and diet-derived fungal xenosiderophores promote salmonella gastrointestinal colonization. Nat Microbiol. 2022;7(12):2025–2038. doi:10.1038/s41564-022-01267-w. 36411353 PMC11981548

[55] Fiers WD, Gao IH, Iliev ID. Gut mycobiota under scrutiny: fungal symbionts or environmental transi ents? Curr Opin Microbiol. 2019;50:79–86. doi:10.1016/j.mib.2019.09.010. 31726316 PMC6908457

[56] Hoffmann C, Dollive S, Grunberg S, Chen J, Li H, Wu GD, Lewis JD, Bushman FD, Pan C. Archaea and fungi of the human gut microbiome: correlations with diet and bacterial residents. PLOS ONE. 2013;8(6):e66019. doi:10.1371/journal.pone.0066019. 23799070 PMC3684604

[57] Heisel T, Montassier E, Johnson A, Al-Ghalith G, Lin Y-W, Wei L-N, Knights D, Gale CA, Krajmalnik-Brown R. High-fat diet changes fungal microbiomes and interkingdom relationship s in the murine gut. mSphere. 2017;2(5). doi:10.1128/mSphere.00351-17. PMC5636226 29034327

[58] Pareek S, Kurakawa T, Das B, Motooka D, Nakaya S, Rongsen-Chandola T, Goyal N, Kayama H, Dodd D, Okumura R, et al. Comparison of Japanese and Indian intestinal microbiota shows diet-dep endent interaction between bacteria and fungi. Npj Biofilms Microbiomes. 2019;5(1). doi:10.1038/s41522-019-0110-9. PMC6925221 31885873

[59] Kennedy MJ, Volz PA. Effect of various antibiotics on gastrointestinal colonization and dissemination by Candida albicans. Sabouraudia. 1985;23(4):265–273. 3901329 10.1080/00362178585380391

[60] Seelbinder B, Chen J, Brunke S, Vazquez-Uribe R, Santhaman R, Meyer A-C, de Oliveira Lino FS, Chan K-F, Loos D, Imamovic L , et al. Antibiotics create a shift from mutualism to competition in human gut communities with a longer-lasting impact on fungi than bacteria. Microbiome. 2020;8(1):133. 32919472 10.1186/s40168-020-00899-6 PMC7488854

[61] Samonis G, Gikas A, Anaissie EJ, Vrenzos G, Maraki S, Tselentis Y, Bodey GP. Prospective evaluation of effects of broad-spectrum antibiotics on gas trointestinal yeast colonization of humans. Antimicrob Agents Chemother. 1993;37(1):51–53. 8431017 10.1128/aac.37.1.51 PMC187603

[62] Clark JD. Influence of antibiotics or certain intestinal bacteria on orally admi nistered Candida albicans in germ-free and conventional mice. Infect Immun. 1971;4(6):731–737. 5005314 10.1128/iai.4.6.731-737.1971 PMC416382

[63] Krause R, Reisinger EC. Candida and antibiotic-associated diarrhoea. Clin Microbiol And Infect. 2005;11(1):1–2. doi:10.1111/j.1469-0691.2004.00978.x. 15649296

[64] Heng X, Jiang Y, Chu W. Influence of Fluconazole Administration on Gut Microbiome, Intestinal Barrier, and Immune Response in Mice. Antimicrob Agents Chemother. 2021;65(6):e02552–02520. 33722893 10.1128/AAC.02552-20 PMC8315907

[65] Bohner F, Papp C, Gácser A. The effect of antifungal resistance development on the virulence of Candida species. FEMS Yeast Res. 2022;22(1):foac019. 35325128 10.1093/femsyr/foac019 PMC9466593

[66] Gulati M, Nobile CJ. Candida albicans biofilms: development, regulation, and molecular mechanisms. Microbes And Infect. 2016;18(5):310–321. doi:10.1016/j.micinf.2016.01.002. 26806384 PMC4860025

[67] Willis KA, Purvis JH, Myers ED, Aziz MM, Karabayir I, Gomes CK, Peters BM, Akbilgic O, Talati AJ, Pierre JF. Fungi form interkingdom microbial communities in the primordial human gut that develop with gestational age. The FASEB J. 2019;33(11):12825. doi:10.1096/fj.201901436RR. 31480903 PMC6902694

[68] Blaser MJ, Devkota S, Kd M, Relman DA, Yassour M, Vbjm Y. Lessons learned from the prenatal microbiome controversy. Microbiome. 2021;9:1–7. 33436098 10.1186/s40168-020-00946-2 PMC7805060

[69] Nagata R, Nagano H, Ogishima D, Nakamura Y, Hiruma M, Sugita T. Transmission of the major skin microbiota, Malassezia, from mot her to neonate. Pediatrics Int. 2012;54(3):350–355. doi:10.1111/j.1442-200X.2012.03563.x. 22300401

[70] Boix-Amorós A, Martinez-Costa C, Querol A, Collado MC, Mira A. Multiple approaches detect the presence of fungi in human breast milk samples from healthy mothers. Sci Rep. 2017;7(1):13016. doi:10.1038/s41598-017-13270-x. 29026146 PMC5638952

[71] Bliss JM, Basavegowda KP, Watson WJ, Sheikh AU, Ryan RM. Ryan RMJTPidj: vertical and horizontal transmission of Candida albicans in very low birth weight infants using DNA fingerprinting techniques. The Pediatr Infect Disease J. 2008;27(3):231–235. doi:10.1097/INF.0b013e31815bb69d. 18277930

[72] Fu Y, Gou W, Wu P, Lai Y, Liang X, Zhang K, Shuai M, Tang J, Miao Z, Chen J, et al. Landscape of the gut mycobiome dynamics during pregnancy and its relat ionship with host metabolism and pregnancy health. Gut. 2024;73(8):1302–1312. 10.1136/gutjnl-2024-332260 PMC11287620 38724219

[73] Juyal D, Sharma M, Pal S, Rathaur VK. Sharma NJNAjoms: emergence of non-albicans candida species in neonatal candidemia. North American Journal of Medical Sciences. 2013;5(9):541. 24251272 10.4103/1947-2714.118919 PMC3818827

[74] Nash AK, Auchtung TA, Wong MC, Smith DP, Gesell JR, Ross MC, Stewart CJ, Metcalf GA, Muzny DM, Gibbs RA, et al. The gut mycobiome of the human microbiome project healthy cohort. Microbiome. 2017;5(1):153. doi:10.1186/s40168-017-0373-4. 29178920 PMC5702186

[75] Kapitan M, Niemiec MJ, Steimle A, Frick JS, Jacobsen ID. Fungi as part of the microbiota and interactions with intestinal bacteria. In: Rodrigues M, editor. Fungal physiology and Immunopathogenesis. ed. Cham: Springer International Publishing; 2019. p. 265–301. 10.1007/82_2018_117 30062595

[76] O’Gorman CM, Fuller HT. Prevalence of culturable airborne spores of selected allergenic and pa thogenic fungi in outdoor air. Atmos Environ. 2008;42(18):4355–4368. doi:10.1016/j.atmosenv.2008.01.009.

[77] Mortensen KL, Mellado E, Lass-Forl C, Rodriguez-Tudela JL, Johansen HK, Arendrup MC. Environmental study of azole-resistant aspergillus fumigatus and other aspergilli in Austria, Denmark, and Spain. Antimicrob Agents Chemother. 2010;54(11):4545–4549. doi:10.1128/AAC.00692-10. 20805399 PMC2976122

[78] Martino C, Dilmore AH, Burcham ZM, Metcalf JL, Jeste D, Rjnrm K. Microbiota succession throughout life from the cradle to the grave. Nature reviews. Microbiology. 2022;20(12):707–720. 35906422 10.1038/s41579-022-00768-z PMC12875531

[79] Shuai M, Fu Y, H-L Z, Gou W, Jiang Z, Liang Y, Miao Z, J-J X, Huynh T, Mljg W, et al. Mapping the human gut mycobiome in middle-aged and elderly adults: multiomics insights and implications for host metabolic health. Gut. 2022;71(9):1812–1820. doi:10.1136/gutjnl-2021-326298. 35017200 PMC9380515

[80] Angebault C, Djossou F, Abélanet S, Permal E, Ben Soltana M, Diancourt L, Bouchier C, Woerther P-L, Catzeflis F, Andremont A, et al. Candida albicans is not always the preferential yeast colonizing human s: a study in Wayampi amerindians. J Infect Dis. 2013;208(10):1705–1716. doi:10.1093/infdis/jit389. 23904289

[81] Sun Y, Zuo T, Cheung CP, Gu W, Wan Y, Zhang F, Chen N, Zhan H, Yeoh YK, Niu J, et al. Population-level configurations of gut mycobiome across 6 ethnicities in urban and rural China. Gastroenterology. 2021;160(1):272–286.e211. doi:10.1053/j.gastro.2020.09.014. 32956679

[82] Mar Rodríguez M, Pérez D, Javier Chaves F, Esteve E, Marin-Garcia P, Xifra G, Vendrell J, Jové M, Pamplona R, Ricart W, et al. Obesity changes the human gut mycobiome. Sci Rep. 2015;5(1). doi:10.1038/srep14600. PMC4600977 26455903

[83] Jj L, Tang J, Li D, Aj W, Ks M, Funari V, Gargus M, Nguyen C, Sharma P, Vi M, et al. Malassezia is associated with Crohn’s disease and exacerbates colitis in mouse models. Cell Host & Microbe. 2019;25(3):377–388.e376. doi:10.1016/j.chom.2019.01.007. 30850233 PMC6417942

[84] Strati F, Di Paola M, Stefanini I, Albanese D, Rizzetto L, Lionetti P, Calabrò A, Jousson O, Donati C, Cavalieri D, et al. Age and gender affect the composition of fungal population of the human gastrointestinal tract. Front Microbiol. 2016;7:1227. doi:10.3389/fmicb.2016.01227. 27536299 PMC4971113

[85] Belkaid Y, Harrison OJ. Homeostatic immunity and the microbiota. Immunity. 2017;46(4):562–576. 28423337 10.1016/j.immuni.2017.04.008 PMC5604871

[86] Bacher P, Hohnstein T, Beerbaum E, Röcker M, Blango MG, Kaufmann S, Röhmel J, Eschenhagen P, Grehn C, Seidel K, et al. Human anti-fungal Th17 immunity and pathology rely on cross-reactivity against Candida albicans. Cell. 2019;176(6):1340–1355.e 1315. 10.1016/j.cell.2019.01.041. 30799037

[87] Kanj AN, Kottom TJ, Schaefbauer KJ, Choudhury M, Limper AH, Skalski JH. Dysbiosis of the intestinal fungal microbiota increases lung resident group 2 innate lymphoid cells and is associated with enhanced asthma severity in mice and humans. Respir Res. 2023;24(1):144. doi:10.1186/s12931-023-02422-5. 37259076 PMC10230676

[88] Shiao SL, Kershaw KM, Limon JJ, You S, Yoon J, Ko EY, Guarnerio J, Potdar AA, McGovern DPB, Bose S, et al. Commensal bacteria and fungi differentially regulate tumor responses to radiation therapy. Cancer Cell 2021, 2021;39(9):1202–1213.e 1206. 10.1016/j.ccell.2021.07.002. 34329585 PMC8830498

[89] Doron I, Leonardi I, Li F XV, Semon WD, A B-D, Gao M, Migaud M, Lin IH, Wy KT, Kusakabe T, et al. Human gut mycobiota tune immunity via CARD9-dependent induction of anti-fungal IgG antibodies. Cell. 2021;184(4):1017–1031.e 1014. 10.1016/j.cell.2021.01.016. 33548172 PMC7936855

[90] Liu YT, Li YQ, Wang YZ. Protective effect of saccharomyces boulardii against intestinal mucosal barrier injury in rats with nonalcoholic fatty liver disease]. Zhonghua gan zang bing za zhi = Zhonghua ganzangbing zazhi =. Chin J Hepatol. 2016;24(12):921–926. doi:10.3760/cma.j.issn.1007-3418.2016.12.009. PMC12769122 28073414

[91] Wang Y, Ren Y, Huang Y, Yu X, Yang Y, Wang D, Shi L, Tao K, Wang G, Wu K. Fungal dysbiosis of the gut microbiota is associated with colorectal cancer in Chinese patients. Am J Transl Res. 2021;13(10):11287–11301. 34786058 PMC8581944

[92] Zhang Z, Li J, Zheng W, Zhao G, Zhang H, Wang X, Guo Y, Qin C, Shi Y. Peripheral lymphoid volume expansion and maintenance are controlled by gut microbiota via RALDH+ dendritic cells. Immunity. 2016;44(2):330–342. doi:10.1016/j.immuni.2016.01.004. 26885858 PMC4757854

[93] Sun S, Sun L, Wang K, Qiao S, Zhao X, Hu X, Chen W, Zhang S, Li H, Dai H, et al. The gut commensal fungus, Candida parapsilosis, promotes high fat-diet induced obesity in mice. Commun Biol. 2021;4(1):1220. doi:10.1038/s42003-021-02753-3. 34697386 PMC8546080

[94] Leonardi I, Gao IH, Lin WY, Allen M, Li F XV, De Celie WD, Putzel MB, Yantiss GG, Rk JM, Johncilla M, et al. Mucosal fungi promote gut barrier function and social behavior via type 17 immunity. Cell. 2022;185(5):831–846.e814. doi:10.1016/j.cell.2022.01.017. 35176228 PMC8897247

[95] Tso GHW, Reales-Calderon JA, Tan ASM, Sem X, Le GTT, Tan TG, Lai GC, Srinivasan KG, Yurieva M, Liao W, et al. Experimental evolution of a fungal pathogen into a gut symbiont. Sci (New York, NY). 2018;362(6414):589–595. doi:10.1126/science.aat0537. 30385579

[96] Shao TY, Ang WXG, Jiang TT, Huang FS, Andersen H, Kinder JM, Pham G, Burg AR, Ruff B, Gonzalez T, et al. Commensal Candida albicans positively calibrates systemic Th17 immunological responses. Cell Host & Microbe. 2019;25(3):404–417.e406. doi:10.1016/j.chom.2019.02.004. 30870622 PMC6419754

[97] Chen YH, Yeung F, Lacey KA, Zaldana K, Lin JD, Bee GCW, McCauley C, Barre RS, Liang SH, Hansen CB, et al. Rewilding of laboratory mice enhances granulopoiesis and immunity through intestinal fungal colonization. Sci Immunol. 2023;8(84):eadd6910. doi:10.1126/sciimmunol.add6910. 37352372 PMC10350741

[98] Wheeler ML, Limon JJ, Bar AS, Leal CA, Gargus M, Tang J, Brown J, Funari VA, Wang HL, Crother TR, et al. Immunological consequences of intestinal fungal dysbiosis. Cell Host & Microbe. 2016;19(6):865–873. doi:10.1016/j.chom.2016.05.003. 27237365 PMC4900921

[99] Li X, Leonardi I, Semon A, Doron I, Gao IH, Putzel GG, Kim Y, Kabata H, Artis D, Fiers WD, et al. Response to fungal dysbiosis by gut-resident CX3CR1(+) mononuclear phagocytes aggravates allergic airway disease. Cell Host & Microbe. 2018;24(6):847–856.e844. doi:10.1016/j.chom.2018.11.003. 30503509 PMC6292739

[100] Kim YG, Udayanga KG, Totsuka N, Weinberg JB, Núñez G, Shibuya A. Gut dysbiosis promotes M2 macrophage polarization and allergic airway inflammation via fungi-induced PGE₂. Cell Host & Microbe. 2014;15(1):95–102. 24439901 10.1016/j.chom.2013.12.010 PMC3957200

[101] Noverr MC, Noggle RM, Toews GB, Huffnagle GB. Role of antibiotics and fungal microbiota in driving pulmonary allergic responses. Infect Immun. 2004;72(9):4996–5003. 15321991 10.1128/IAI.72.9.4996-5003.2004 PMC517468

[102] Iliev ID, Funari VA, Taylor KD, Nguyen Q, Reyes CN, Strom SP, Brown J, Becker CA, Fleshner PR, Dubinsky M, et al. Interactions between commensal fungi and the C-type lectin receptor dectin-1 influence colitis. Sci (New York, NY). 2012;336(6086):1314–1317. doi:10.1126/science.1221789. PMC3432565 22674328

[103] Lewis JD, Chen EZ, Baldassano RN, Otley AR, Griffiths AM, Lee D, Bittinger K, Bailey A, Friedman ES, Hoffmann C, et al. Inflammation, antibiotics, and diet as environmental stressors of the gut microbiome in pediatric Crohn’s disease. Cell Host & Microbe. 2015;18(4):489–500. 26468751 10.1016/j.chom.2015.09.008 PMC4633303

[104] Liguori G, Lamas B, Richard ML, Brandi G, da Costa G, Hoffmann TW, Di Simone MP, Calabrese C, Poggioli G, Langella P, et al. Fungal dysbiosis in mucosa-associated microbiota of Crohn’s disease patients. J Crohn’s & Colitis. 2016;10(3):296–305. 26574491 10.1093/ecco-jcc/jjv209 PMC4957473

[105] Iliev ID, Leonardi I. Fungal dysbiosis: immunity and interactions at mucosal barriers. Nat Rev Immunol. 2017;17(10):635–646. 28604735 10.1038/nri.2017.55 PMC5724762

[106] Qin X, Gu Y, Liu T, Wang C, Zhong W, Wang B, Cao H. Gut mycobiome: a promising target for colorectal cancer. Biochim Et Biophys Acta (BBA) - Rev On Cancer. 2021;1875(1):188489. doi:10.1016/j.bbcan.2020.188489. 33278512

[107] Bowman SM, Free SJ. The structure and synthesis of the fungal cell wall. BioEssays. 2006;28(8):799–808. doi:10.1002/bies.20441. 16927300

[108] Kashem SW, Igyarto BZ, Gerami-Nejad M, Kumamoto Y, Mohammed JA, Jarrett E, Drummond RA, Zurawski SM, Zurawski G, Berman J, et al. Candida albicans morphology and dendritic cell subsets determine T helper cell differentiation. Immunity. 2015;42(2):356–366. doi:10.1016/j.immuni.2015.01.008. 25680275 PMC4343045

[109] Underhill DM, Pearlman E. Immune interactions with pathogenic and commensal fungi: a two-way street. Immunity. 2015;43(5):845–858. doi:10.1016/j.immuni.2015.10.023. 26588778 PMC4865256

[110] Plato A, Hardison SE, Brown GD. Pattern recognition receptors in antifungal immunity. Semin Immunopathology. 2015;37(2):97–106. 10.1007/s00281-014-0462-4 PMC4326652 25420452

[111] Brown GD, Gordon S. Fungal β-Glucans and mammalian immunity. Immunity. 2003;19(3):311–315. doi:10.1016/S1074-7613(03)00233-4. 14499107

[112] Taylor PR, Tsoni SV, Willment JA, Dennehy KM, Rosas M, Findon H, Haynes K, Steele C, Botto M, Gordon S, et al. Dectin-1 is required for β-glucan recognition and control of fungal infection. Nat Immunol. 2007;8(1):31–38. doi:10.1038/ni1408. 17159984 PMC1888731

[113] Hise AG, Tomalka J, Ganesan S, Patel K, Hall BA, Brown GD, Fitzgerald KA. An essential role for the NLRP3 inflammasome in host defense against the human fungal pathogen Candida albicans. Cell Host & Microbe. 2009;5(5):487–497. doi:10.1016/j.chom.2009.05.002. 19454352 PMC2824856

[114] Gessner MA, Werner JL, Lilly LM, Nelson MP, Metz AE, Dunaway CW, Chan YR, Ouyang W, Brown GD, Weaver CT, et al. Dectin-1-dependent interleukin-22 contributes to early innate lung defense against aspergillus fumigatus. Infect Immun. 2012;80(1):410–417. 22038916 10.1128/IAI.05939-11 PMC3255669

[115] Ferwerda B, Ferwerda G, Plantinga TS, Willment JA, van Spriel AB, Venselaar H, Elbers CC, Johnson MD, Cambi A, Huysamen C, et al. Human dectin-1 deficiency and mucocutaneous fungal infections. The N Engl J Med. 2009;361(18):1760–1767. 19864674 10.1056/NEJMoa0901053 PMC2773015

[116] Robinson MJ, Osorio F, Rosas M, Freitas RP, Schweighoffer E, Gross O, Verbeek JS, Ruland J, Tybulewicz V, Brown GD, et al. Dectin-2 is a Syk-coupled pattern recognition receptor crucial for Th17 responses to fungal infection. The J Exp Med. 2009;206(9):2037–2051. doi:10.1084/jem.20082818. 19703985 PMC2737172

[117] Zhu L-L, Zhao X-Q, Jiang C, You Y, Chen X-P, Jiang Y-Y, Jia X-M, Lin X. C-Type lectin receptors dectin-3 and dectin-2 form a heterodimeric pat tern-recognition receptor for Host defense against fungal infection. Immunity. 2013;39(2):324–334. doi:10.1016/j.immuni.2013.05.017. 23911656

[118] Wüthrich M, Wang H, Li M, Lerksuthirat T, Hardison SE, Brown GD, Klein B. Fonsecaea pedrosoi-induced Th17-cell differentiation in mice is fostered by dectin-2 and suppressed by mincle recognition. Eur J Immunol. 2015;45(9):2542–2552. doi:10.1002/eji.201545591. 26140582 PMC4562893

[119] Nakamura Y, Sato K, Yamamoto H, Matsumura K, Matsumoto I, Nomura T, Miyasaka T, Ishii K, Kanno E, Tachi M, et al. Dectin-2 deficiency promotes Th2 response and mucin production in the lungs after pulmonary infection with Cryptococcus neoformans. Infect Immun. 2015;83(2):671–681. doi:10.1128/IAI.02835-14. 25422263 PMC4294247

[120] Zhao XQ, Zhu LL, Chang Q, Jiang C, You Y, Luo T, Jia XM, Lin X. C-type lectin receptor dectin-3 mediates trehalose 6,6′-dimycolate (TDM)-induced mincle expression through CARD9/Bcl10/MALT1-dependent nuclear factor (NF)-κB activation. The J Biol Chem. 2014;289(43):30052–30062. doi:10.1074/jbc.M114.588574. 25202022 PMC4208012

[121] Zhu LL, Zhao XQ, Jiang C, You Y, Chen XP, Jiang YY, Jia XM, Lin X. C-type lectin receptors dectin-3 and dectin-2 form a heterodimeric pattern-recognition receptor for host defense against fungal infection. Immunity. 2013;39(2):324–334. doi:10.1016/j.immuni.2013.05.017. 23911656

[122] Wang T, Pan D, Zhou Z, You Y, Jiang C, Zhao X, Lin X, Gaffen SL. Dectin-3 deficiency promotes colitis development due to impaired antifungal innate immune responses in the gut. PLOS Pathog. 2016;12(6):e1005662. doi:10.1371/journal.ppat.1005662. 27280399 PMC4900642

[123] Ishikawa T, Itoh F, Yoshida S, Saijo S, Matsuzawa T, Gonoi T, Saito T, Okawa Y, Shibata N, Miyamoto T, et al. Identification of distinct ligands for the C-type lectin receptors mincle and dectin-2 in the pathogenic fungus malassezia. Cell Host & Microbe. 2013;13(4):477–488. 23601109 10.1016/j.chom.2013.03.008

[124] Lamas B, Richard ML, Leducq V, Pham HP, Michel ML, Da Costa G, Bridonneau C, Jegou S, Hoffmann TW, Natividad JM, et al. CARD9 impacts colitis by altering gut microbiota metabolism of tryptophan into aryl hydrocarbon receptor ligands. Nat Med. 2016;22(6):598–605. doi:10.1038/nm.4102. 27158904 PMC5087285

[125] Zelante T, Iannitti RG, Cunha C, De Luca A, Giovannini G, Pieraccini G, Zecchi R, D’Angelo C, Massi-Benedetti C, Fallarino F, et al. Tryptophan catabolites from microbiota engage aryl hydrocarbon receptor and balance mucosal reactivity via interleukin-22. Immunity. 2013;39(2):372–385. doi:10.1016/j.immuni.2013.08.003. 23973224

[126] Wang T, Fan C, Yao A, Xu X, Zheng G, You Y, Jiang C, Zhao X, Hou Y, Hung MC, et al. The adaptor protein CARD9 protects against colon cancer by restricting Mycobiota-mediated expansion of myeloid-derived suppressor cells. Immunity. 2018;49(3):504–514.e4. doi:10.1016/j.immuni.2018.08.018. 30231984 PMC6880241

[127] Malik A, Sharma D, Malireddi RKS, Guy CS, Chang TC, Olsen SR, Neale G, Vogel P, Kanneganti TD. SYK-CARD9 signaling axis promotes gut fungi-mediated inflammasome activation to restrict colitis and colon cancer. Immunity. 2018;49(3):515–530.e515. doi:10.1016/j.immuni.2018.08.024. 30231985 PMC6541497

[128] Chiba S, Ikushima H, Ueki H, Yanai H, Kimura Y, Hangai S, Nishio J, Negishi H, Tamura T, Saijo S, et al. Recognition of tumor cells by dectin-1 orchestrates innate immune cell s for anti-tumor responses. eLife. 2014;3:3. doi:10.7554/eLife.04177. PMC4161974 25149452

[129] Daley D, Mani VR, Mohan N, Akkad N, Ochi A, Heindel DW, Lee KB, Zambirinis CP, Pandian GSDB, Savadkar S, et al. Dectin 1 activation on macrophages by galectin 9 promotes pancreatic c arcinoma and peritumoral immune tolerance. Nat Med. 2017;23(5):556–567. doi:10.1038/nm.4314. 28394331 PMC5419876

[130] Kimura Y, Inoue A, Hangai S, Saijo S, Negishi H, Nishio J, Yamasaki S, Iwakura Y, Yanai H, Taniguchi T. The innate immune receptor dectin-2 mediates the phagocytosis of cancer cells by Kupffer cells for the suppression of liver metastasis. Proc Natl Acad Sci USA. 2016;113(49):14097–14102. doi:10.1073/pnas.1617903113. 27872290 PMC5150405

[131] Zhu Y, Shi T, Lu X, Xu Z, Qu J, Zhang Z, Shi G, Shen S, Hou Y, Chen Y, et al. Fungal-induced glycolysis in macrophages promotes colon cancer by enha ncing innate lymphoid cell secretion of IL-22. Embo J. 2021;40(11):e105320. 33591591 10.15252/embj.2020105320 PMC8167358

[132] Gadaleta RM, van Erpecum KJ, Oldenburg B, Willemsen EC, Renooij W, Murzilli S, Klomp LW, Siersema PD, Schipper ME, Danese S, et al. Farnesoid X receptor activation inhibits inflammation and preserves the intestinal barrier in inflammatory bowel disease. Gut. 2011;60(4):463–472. doi:10.1136/gut.2010.212159. 21242261

[133] Briard B, Fontaine T, Samir P, Place DE, Muszkieta L, Malireddi RKS, Karki R, Christgen S, Bomme P, Vogel P, et al. Galactosaminogalactan activates the inflammasome to provide host prote ction. Nature. 2020;588(7839):688–692. doi:10.1038/s41586-020-2996-z. 33268895 PMC8086055

[134] Tomalka J, Ganesan S, Azodi E, Patel K, Majmudar P, Hall BA, Fitzgerald KA, Hise AG, Filler SG. A novel role for the NLRC4 inflammasome in mucosal defenses against the fungal pathogen Candida albicans. PLOS Pathog. 2011;7(12):e1002379. doi:10.1371/journal.ppat.1002379. 22174673 PMC3234225

[135] Borghi M, De Luca A, Puccetti M, Jaeger M, Mencacci A, Oikonomou V, Pariano M, Garlanda C, Moretti S, Bartoli A, et al. Pathogenic NLRP3 inflammasome activity during Candida infection is negatively regulated by IL-22 via activation of NLRC4 and IL-1Ra. Cell host & microbe 2015, Cell Host & Microbe. 2015;18(2):198–209. doi:10.1016/j.chom.2015.07.004. 26269955

[136] Joly S, Ma N, Sadler JJ, Soll DR, Cassel SL, Sutterwala FS. Cutting edge: Candida albicans hyphae formation triggers activation of the NLRP3 inflammasome. J Immunol. 2009;183(6):3578–3581. doi:10.4049/jimmunol.0901323. 19684085 PMC2739101

[137] Kasper L, König A, Koenig PA, Gresnigt MS, Westman J, Drummond RA, Lionakis MS, Groß O, Ruland J, Naglik JR, et al. The fungal peptide toxin Candidalysin activates the NLRP3 inflammasome and causes cytolysis in mononuclear phagocytes. Nat Commun. 2018;9(1):4260. doi:10.1038/s41467-018-06607-1. 30323213 PMC6189146

[138] Zhao X, Guo Y, Jiang C, Chang Q, Zhang S, Luo T, Zhang B, Jia X, Hung MC, Dong C, et al. JNK1 negatively controls antifungal innate immunity by suppressing CD23 expression. Nat Med. 2017;23(3):337–346. doi:10.1038/nm.4260. 28112734 PMC5592785

[139] Li K, Chatterjee A, Qian C, Lagree K, Wang Y, Becker CA, Freeman MR, Murali R, Yang W, Underhill DM. Profiling phagosome proteins identifies PD-L1 as a fungal-binding receptor. Nature. 2024;630(8017):736–743. doi:10.1038/s41586-024-07499-6. 38839956 PMC12755075

[140] Jouault T, Ibata‐Ombetta S, Takeuchi O, Trinel PA, Sacchetti P, Lefebvre P, Akira S, Poulain D. Candida albicans phospholipomannan is sensed through toll-like receptors. J Infect Dis. 2003;188(1):165–172. doi:10.1086/375784. 12825186

[141] Stephen-Victor E, Karnam A, Fontaine T, Beauvais A, Das M, Hegde P, Prakhar P, Holla S, Balaji KN, Kaveri SV, et al. Aspergillus fumigatus cell Wall α-(1,3)-glucan stimulates regulatory T -Cell polarization by inducing PD-L1 expression on human dendritic cel ls. J Infect Dis. 2017;216(10):1281–1294. doi:10.1093/infdis/jix469. 28968869

[142] Netea MG. Immune sensing of Candida albicans requires cooperative recognition of mannans and glucans by lectin and Toll-like receptors. J Clin Investigation. 2006;116(6):1642–1650. doi:10.1172/JCI27114. PMC1462942 16710478

[143] Mambula SS, Sau K, Henneke P, Golenbock DT, Levitz SM. Toll-like receptor (TLR) signaling in response to Aspergillus fumigatus. J Biol Chem. 2002;277(42):39320–39326. doi:10.1074/jbc.M201683200. 12171914

[144] Plantinga TS, Johnson MD, Scott WK, van de Vosse E, Velez Edwards DR, Smith PB, Alexander BD, Yang JC, Kremer D, Laird GM, et al. Toll-like receptor 1 polymorphisms increase susceptibility to candidem IA. J Infect Dis. 2012;205(6):934–943. doi:10.1093/infdis/jir867. 22301633 PMC3282566

[145] Skevaki C, Pararas M, Kostelidou K, Tsakris A, Routsias JG. Single nucleotide polymorphisms of Toll-like receptors and susceptibility to infectious diseases. Clin And Exp Immunol. 2015;180(2):165–177. doi:10.1111/cei.12578. 25560985 PMC4408151

[146] Takeuchi O, Akira S. Pattern recognition receptors and inflammation. Cell. 2010;140(6):805–820. doi:10.1016/j.cell.2010.01.022. 20303872

[147] Krüger W, Vielreicher S, Kapitan M, Jacobsen ID, Niemiec MJ. Fungal-bacterial interactions in health and disease. Fungal-Bacterial Interact In Health And Disease Pathog (Basel, Switzerland) 2019;8(2):70. doi:10.3390/pathogens8020070. PMC6630686 31117285

[148] Fan D, Coughlin LA, Neubauer MM, Kim J, Kim MS, Zhan X, Simms-Waldrip TR, Xie Y, Hooper LV, Koh AY. Activation of HIF-1α and LL-37 by commensal bacteria inhibits Candida albicans colonization. Nat Med. 2015;21(7):808–814. doi:10.1038/nm.3871. 26053625 PMC4496259

[149] Alonso-Roman R, Last A, Mirhakkak MH, Sprague JL, Möller L, Großmann P, Graf K, Gratz R, Mogavero S, Vylkova S, et al. Lactobacillus rhamnosus colonisation antagonizes Candida albicans by forcing metabolic adaptations that compromise pathogenicity. Nat Commun. 2022;13(1):3192. doi:10.1038/s41467-022-30661-5. 35680868 PMC9184479

[150] Gu P, Liu R, Yang Q, Xie L, Wei R, Li J, Mei F, Chen T, Zeng Z, He Y, et al. A metabolite from commensal Candida albicans enhances the bactericidal activity of macrophages and protects against sepsis. Cellular & Mol Immunol. 2023;20(10):1156–1170. doi:10.1038/s41423-023-01070-5. 37553429 PMC10541433

[151] Cuskin F, Lowe EC, Temple MJ, Zhu Y, Cameron E, Pudlo NA, Porter NT, Urs K, Thompson AJ, Cartmell A, et al. Human gut bacteroidetes can utilize yeast mannan through a selfish mechanism. Nature. 2015;517(7533):165–169. doi:10.1038/nature13995. 25567280 PMC4978465

[152] Manners DJ, Masson AJ, Patterson JC, Björndal H, Lindberg B. The structure of a β-(1→6)- d -glucan from yeast cell walls. The Biochemical J. 1973;135(1):31–36. doi:10.1042/bj1350031. PMC1165785 4590991

[153] Li S, Yu X, Wu W, Chen DZ, Xiao M, Huang X. RETRACTED: the opportunistic human fungal pathogen Candida albicans promotes the growth and proliferation of commensal Escherichia coli through an iron-responsive pathway. Microbiological Res. 2018;207:232–239. doi:10.1016/j.micres.2017.12.008. 29458859

[154] van Leeuwen Pt, van der Peet Jm, Bikker FJ, Hoogenkamp MA, Oliveira Paiva AM, Kostidis S, Mayboroda OA, Smits WK, Krom BP, van Leeuwen PT, et al. Interspecies interactions between Clostridium difficile and Candida albicans. mSphere. 2016;1(6). doi:10.1128/mSphere.00187-16. PMC5103046 27840850

[155] Lambooij JM, Hoogenkamp MA, Brandt BW, Janus MM, Krom BP. Fungal mitochondrial oxygen consumption induces the growth of strict anaerobic bacteria. Fungal Genet Biol. 2017;109:1–6. doi:10.1016/j.fgb.2017.10.001. 28989089

[156] Zuo T, Wong SH, Cheung CP, Lam K, Lui R, Cheung K, Zhang F, Tang W, Ching JYL, Jcy W, et al. Gut fungal dysbiosis correlates with reduced efficacy of fecal microbiota transplantation in clostridium difficile infection. Nat Commun. 2018;9(1):3663. doi:10.1038/s41467-018-06103-6. 30202057 PMC6131390

[157] Rakoff-Nahoum S, Paglino J, Eslami-Varzaneh F, Edberg S, Medzhitov R. Recognition of commensal microflora by toll-like receptors is required for intestinal homeostasis. Cell. 2004;118(2):229–241. doi:10.1016/j.cell.2004.07.002. 15260992

[158] Sendid B, Jouault T, Vitse A, Fradin C, Colombel JF, Poulain D. Glycannes pariétaux de levures et anticorps spécifiques. Medecine Sci: M/S. 2009;25(5):473–481. doi:10.1051/medsci/2009255473. 19480828

[159] Li Q, Wang C, Tang C, He Q, Li N, Li J. Dysbiosis of gut fungal microbiota is associated with mucosal inflamma tion in Crohn’s disease. J Clin Gastroenterol. 2014;48(6):513–523. doi:10.1097/MCG.0000000000000035. 24275714 PMC4059552

[160] Lewis JD, Chen EZ, Baldassano RN, Otley AR, Griffiths AM, Lee D, Bittinger K, Bailey A, Friedman ES, Hoffmann C, et al. Inflammation, antibiotics, and diet as environmental stressors of the gut microbiome in pediatric Crohn’s disease. Cell Host & Microbe. 2015;18(4):489–500. doi:10.1016/j.chom.2015.09.008. 26468751 PMC4633303

[161] Sokol H, Leducq V, Aschard H, Pham HP, Jegou S, Landman C, Cohen D, Liguori G, Bourrier A, Nion-Larmurier I, et al. Fungal microbiota dysbiosis in IBD. Gut. 2017;66(6):1039–1048. doi:10.1136/gutjnl-2015-310746. 26843508 PMC5532459

[162] Zhu Y, Shi T, Lu X, Xu Z, Qu J, Zhang Z, Shi G, Shen S, Hou Y, Chen Y, et al. Fungal-induced glycolysis in macrophages promotes colon cancer by enhancing innate lymphoid cell secretion of IL-22. The EMBO J. 2021;40(11):e105320. doi:10.15252/embj.2020105320. 33591591 PMC8167358

[163] You N, Xu J, Wang L, Zhuo L, Zhou J, Song Y, Ali A, Luo Y, Yang J, Yang W, et al. Fecal fungi dysbiosis in nonalcoholic fatty liver disease. Obesity. 2021;29(2):350–358. doi:10.1002/oby.23073. 33491316

[164] Demir M, Lang S, Hartmann P, Duan Y, Martin A, Miyamoto Y, Bondareva M, Zhang X, Wang Y, Kasper P, et al. The fecal mycobiome in non-alcoholic fatty liver disease. J Hepatol. 2022;76(4):788–799. doi:10.1016/j.jhep.2021.11.029. 34896404 PMC8981795

[165] Yang AM, Inamine T, Hochrath K, Chen P, Wang L, Llorente C, Bluemel S, Hartmann P, Xu J, Koyama Y, et al. Intestinal fungi contribute to development of alcoholic liver disease. The J Clin Investigation. 2017;127(7):2829–2841. doi:10.1172/JCI90562. PMC5490775 28530644

[166] Lang S, Duan Y, Liu J, Torralba MG, Kuelbs C, Ventura-Cots M, Abraldes JG, Bosques-Padilla F, Verna EC, Brown RS Jr., et al. Intestinal fungal dysbiosis and systemic immune response to fungi in P atients with alcoholic hepatitis. Hepatology. 2020;71(2):522–538. doi:10.1002/hep.30832. 31228214 PMC6925657

[167] Bajaj JS, Liu EJ, Kheradman R, Fagan A, Heuman DM, White M, Gavis EA, Hylemon P, Sikaroodi M, Gillevet PM. Fungal dysbiosis in cirrhosis. Gut. 2018;67(6):1146–1154. doi:10.1136/gutjnl-2016-313170. 28578302

[168] Jiang S, Xu L, Chen Y, Shu Z, Lv L, Zhao Y, Bi K, Yang S, Wang Q, Li L. Longitudinal gut fungal alterations and potential fungal biomarkers for the progression of primary liver disease. Sci China Life Sci. 2024;67(6):1183–1198. doi:10.1007/s11427-023-2458-1. 38413553

[169] Chen Y, Chen Z, Guo R, Chen N, Lu H, Huang S, Wang J, Li L. Correlation between gastrointestinal fungi and varying degrees of chronic hepatitis B virus infection. Diagn Microbiol Infect Dis. 2011;70(4):492–498. doi:10.1016/j.diagmicrobio.2010.04.005. 20846815

[170] Kochar B, Orkaby AR, Ananthakrishnan AN, Ritchie CS. Frailty in inflammatory bowel diseases: an emerging concept. Ther Adv Gastroenterol 2021. 2021;14:17562848211025474. doi:10.1177/17562848211025474. PMC8477705 34594400

[171] Sartor RB, Wu GD. Roles for intestinal bacteria, viruses, and fungi in pathogenesis of Inflammatory bowel diseases and therapeutic approaches. Gastroenterology. 2017;152(2):327–339.e324. doi:10.1053/j.gastro.2016.10.012. 27769810 PMC5511756

[172] Beheshti-Maal A, Shahrokh S, Ansari S, Mirsamadi ES, Yadegar A, Mirjalali H, Zali MR. Gut mycobiome: the probable determinative role of fungi in IBD patients. Mycoses. 2021;64(5):468–476. doi:10.1111/myc.13238. 33421192

[173] Nilsson RH, Ryberg M, Kristiansson E, Abarenkov K, Larsson K-H, Kõljalg U, Fairhead C. Taxonomic reliability of DNA sequences in public sequence databases: a fungal perspective. PLOS ONE. 2006;1(1):e59. doi:10.1371/journal.pone.0000059. 17183689 PMC1762357

[174] Chehoud C, Albenberg LG, Judge C, Hoffmann C, Grunberg S, Bittinger K, Baldassano RN, Lewis JD, Bushman FD, Wu GD. Fungal signature in the gut microbiota of pediatric patients with inflammatory bowel disease. Inflamm Bowel Dis. 2015;21(8):1948–1956. doi:10.1097/MIB.0000000000000454. 26083617 PMC4509842

[175] Caetano CF, Gaspar C, Martinez-de-Oliveira J, Palmeira-de-Oliveira A, Rolo J. The role of yeasts in human health: a review. Life (Basel). 2023;13(4):924. doi:10.3390/life13040924. 37109452 PMC10143383

[176] Hoarau G, Mukherjee PK, Gower-Rousseau C, Hager C, Chandra J, Retuerto MA, Neut C, Vermeire S, Clemente J, Colombel JF, et al. Bacteriome and mycobiome interactions underscore microbial dysbiosis in familial Crohn’s disease. mBio. 2016;7(5). doi:10.1128/mBio.01250-16. PMC5030358 27651359

[177] Torres J, Petralia F, Sato T, Wang P, Telesco SE, Choung RS, Strauss R, Li XJ, Laird RM, Gutierrez RL, et al. Serum biomarkers identify patients who will develop inflammatory bowel diseases up to 5 years before diagnosis. Gastroenterology. 2020;159(1):96–104. doi:10.1053/j.gastro.2020.03.007. 32165208

[178] Ott SJ, Kühbacher T, Musfeldt M, Rosenstiel P, Hellmig S, Rehman A, Drews O, Weichert W, Timmis KN, Schreiber S. Fungi and inflammatory bowel diseases: alterations of composition and diversity. Scand J Gastroenterol. 2008;43(7):831–841. doi:10.1080/00365520801935434. 18584522

[179] Li Q, Wang C, Tang C, He Q, Li N, Li J. Dysbiosis of gut fungal microbiota is associated with mucosal inflammation in Crohn’s disease. J Clin Gastroenterol. 2014;48(6):513–523. doi:10.1097/MCG.0000000000000035. 24275714 PMC4059552

[180] Azcárate-Peril MA, Sikes M, Bruno-Bárcena JM. The intestinal microbiota, gastrointestinal environment and colorectal cancer: a putative role for probiotics in prevention of colorectal cancer? Am J Physiol Gastrointestinal And Liver Physiol. 2011;301(3):G401–424. doi:10.1152/ajpgi.00110.2011. PMC3774253 21700901

[181] Chen W, Liu F, Ling Z, Tong X, Xiang C, Moschetta A. Human intestinal lumen and mucosa-associated microbiota in patients wi th colorectal cancer. PLOS ONE. 2012;7(6):e39743. doi:10.1371/journal.pone.0039743. 22761885 PMC3386193

[182] Candela M, Guidotti M, Fabbri A, Brigidi P, Franceschi C, Fiorentini C. Human intestinal microbiota: cross-talk with the host and its potentia l role in colorectal cancer. Crit Rev Microbiol. 2011;37(1):1–14. doi:10.3109/1040841X.2010.501760. 20874522

[183] Coker OO, Nakatsu G, Dai RZ, Wkk W, Wong SH, Ng SC, Chan FKL, Sung JJY, Yu J. Enteric fungal microbiota dysbiosis and ecological alterations in colorectal cancer. Gut. 2019;68(4):654–662. doi:10.1136/gutjnl-2018-317178. 30472682 PMC6580778

[184] Gao R, Kong C, Huang L, Li H, Qu X, Liu Z, Lan P, Wang J, Qin H. Mucosa-associated microbiota signature in colorectal cancer. Eur J Clin Microbiol & Infect Dis. 2017;36(11):2073–2083. doi:10.1007/s10096-017-3026-4. 28600626

[185] Zhu Y, Shi T, Lu X, Xu Z, Qu J, Zhang Z, Shi G, Shen S, Hou Y, Chen Y, et al. Fungal‐induced glycolysis in macrophages promotes colon cancer by enhancing innate lymphoid cell secretion of IL‐22. Embo J. 2021;40(11):e105320. doi:10.15252/embj.2020105320. 33591591 PMC8167358

[186] Chin S-F, Megat Mohd Azlan PIH, Mazlan L, H-M N. Identification of schizosaccharomyces pombe in the guts of healthy individuals and patients with colorectal cancer: preliminary evidence from a gut microbiome secretome study. Gut Pathog. 2018;10(1). doi:10.1186/s13099-018-0258-5. PMC6040075 30008808

[187] Lin Y, Lau H-H, Liu Y, Kang X, Wang Y, Ting N-N, Kwong T-Y, Han J, Liu W, Liu C, et al. Altered mycobiota signatures and enriched pathogenic aspergillus rambe llii are associated with colorectal cancer based on multicohort fecal metagenomic analyses. Gastroenterology. 2022;163(4):908–921. doi:10.1053/j.gastro.2022.06.038. 35724733

[188] Schnabl B, Brenner DA. Interactions between the intestinal microbiome and liver diseases. Gastroenterology. 2014;146(6):1513–1524. doi:10.1053/j.gastro.2014.01.020. 24440671 PMC3996054

[189] Yang R, Xu Y, Dai Z, Lin X, Wang H. The immunologic role of gut microbiota in patients with chronic HBV in fection. J Immunol Res. 2018;2018:1–6. doi:10.1155/2018/2361963. PMC6083645 30148173

[190] Diehl AM, Day C. Day C: cause, pathogenesis, and treatment of Nonalcoholic Steatohepatitis. The N Engl J Med. 2017;377(21):2063–2072. doi:10.1056/NEJMra1503519. 29166236

[191] Wong VW, Wong GL, Choi PC, Chan AW, Li MK, Chan HY, Chim AM, Yu J, Sung JJ, Chan HL. Disease progression of non-alcoholic fatty liver disease: a prospective study with paired liver biopsies at 3 years. Gut. 2010;59(7):969–974. doi:10.1136/gut.2009.205088. 20581244

[192] Voican CS, Perlemuter G, Naveau S. Mechanisms of the inflammatory reaction implicated in alcoholic hepatitis: 2011 update. Clinics And Res In Hepatol And Gastroenterol. 2011;35(6–7):465–474. doi:10.1016/j.clinre.2011.01.017. 21571602

[193] Hartmann P, Lang S, Zeng S, Duan Y, Zhang X, Wang Y, Bondareva M, Kruglov A, Fouts DE, Stärkel P, et al. Dynamic changes of the fungal microbiome in alcohol use disorder. Front Physiol. 2021;12:699253. doi:10.3389/fphys.2021.699253. 34349667 PMC8327211

[194] Sun S, Wang K, Sun L, Cheng B, Qiao S, Dai H, Shi W, Ma J, Liu H. Therapeutic manipulation of gut microbiota by polysaccharides of Wolfiporia cocos reveals the contribution of the gut fungi-induced PGE2 to alcoholic hepatic steatosis. Gut Microbes. 2020;12(1):1830693. doi:10.1080/19490976.2020.1830693. 33106075 PMC7592601

[195] Zeng S, Hartmann P, Park M, Duan Y, Lang S, Llorente C, Wang Y, Cabré N, Fouts DE, Bacher P, et al. Malassezia restricta promotes alcohol-induced liver injury. Hepatol Commun. 2023;7(2):e0029. doi:10.1097/HC9.0000000000000029. 36706195 PMC9988279

[196] Chu H, Duan Y, Lang S, Jiang L, Wang Y, Llorente C, Liu J, Mogavero S, Bosques-Padilla F, Abraldes JG, et al. The Candida albicans exotoxin candidalysin promotes alcohol-associated liver disease. J Hepatol. 2020;72(3):391–400. doi:10.1016/j.jhep.2019.09.029. 31606552 PMC7031049

[197] Verma AH, Richardson JP, Zhou C, Coleman BM, Moyes DL, Ho J, Huppler AR, Ramani K, McGeachy MJ, Mufazalov IA, et al. Oral epithelial cells orchestrate innate type 17 responses to Candida albicans through the virulence factor candidalysin. Sci Immunol. 2017;2(17). doi:10.1126/sciimmunol.aam8834. PMC5881387 29101209

[198] Viebahn G, Hartmann P, Lang S, Demir M, Zhang X, Fouts DE, Stärkel P, Schnabl B. Fungal signature differentiates alcohol-associated liver disease from nonalcoholic fatty liver disease. Gut Microbes. 2024;16(1):2307586. doi:10.1080/19490976.2024.2307586. 38298161 PMC10841010

[199] Yang A-M, Inamine T, Hochrath K, Chen P, Wang L, Llorente C, Bluemel S, Hartmann P, Xu J, Koyama Y, et al. Intestinal fungi contribute to development of alcoholic liver disease. J Clin Investigation. 2017;127(7):2829–2841. doi:10.1172/JCI90562. PMC5490775 28530644

[200] Singh RK, Chang H-W, Yan D, Lee KM, Ucmak D, Wong K, Abrouk M, Farahnik B, Nakamura M, Zhu TH, et al. Influence of diet on the gut microbiome and implications for human health. J Transl Med. 2017;15(1):73. doi:10.1186/s12967-017-1175-y. 28388917 PMC5385025

[201] van der Merwe M, Sharma S, Caldwell JL, Smith NJ, Gomes CK, Bloomer RJ, Buddington RK, Pierre JF. Time of feeding alters obesity-associated parameters and gut bacterial communities, but not fungal populations, in C57BL/6 male mice. Curr Developments In Nutr. 2020;4(2):nzz145. doi:10.1093/cdn/nzz145. PMC6992463 32025616

[202] Heisel T, Montassier E, Johnson A, Al-Ghalith G, Lin YW, Wei LN, Knights D, Gale CA, Krajmalnik-Brown R. High-fat diet changes fungal microbiomes and interkingdom relationships in the murine gut. mSphere. mSphere. 2017;2(5). doi:10.1128/mSphere.00351-17. PMC5636226 29034327

[203] Wu GD, Chen J, Hoffmann C, Bittinger K, Chen YY, Keilbaugh SA, Bewtra M, Knights D, Walters WA, Knight R, et al. Linking long-term dietary patterns with gut microbial enterotypes. Sci (New York, NY). 2011;334(6052):105–108. doi:10.1126/science.1208344. PMC3368382 21885731

[204] Costabile A, Klinder A, Fava F, Napolitano A, Fogliano V, Leonard C, Gibson GR, Tuohy KM. Whole-grain wheat breakfast cereal has a prebiotic effect on the human gut microbiota: a double-blind, placebo-controlled, crossover study. Br J Nutr. 2008;99(1):110–120. doi:10.1017/S0007114507793923. 17761020

[205] Carvalho-Wells AL, Helmolz K, Nodet C, Molzer C, Leonard C, McKevith B, Thielecke F, Jackson KG, Tuohy KM. Determination of the in vivo prebiotic potential of a maize-based whole grain breakfast cereal: a human feeding study. Br J Nutr. 2010;104(9):1353–1356. doi:10.1017/S0007114510002084. 20487589

[206] De Filippo C, Cavalieri D, Di Paola M, Ramazzotti M, Poullet JB, Massart S, Collini S, Pieraccini G, Lionetti P. Impact of diet in shaping gut microbiota revealed by a comparative study in children from Europe and rural Africa. Proc Natl Acad Sci USA. 2010;107(33):14691–14696. doi:10.1073/pnas.1005963107. 20679230 PMC2930426

[207] Mims TS, Abdallah QA, Stewart JD, Watts SP, White CT, Rousselle TV, Gosain A, Bajwa A, Han JC, Willis KA, et al. The gut mycobiome of healthy mice is shaped by the environment and cor relates with metabolic outcomes in response to diet. Commun Biol. 2021;4(1). doi:10.1038/s42003-021-01820-z. PMC7935979 33674757

[208] Jeziorek M, Frej-Mądrzak M, Choroszy-Król I. The influence of diet on gastrointestinal Candida spp. colonization and the susceptibility of Candida spp. To antifungal drugs. Roczniki Panstwowego Zakladu Higieny. 2019;70(2):195–200. doi:10.32394/rpzh.2019.0070. 31215785

[209] Gunsalus KTW, Tornberg-Belanger SN, Matthan NR, Lichtenstein AH, Kumamoto CA, Mitchell AP. Manipulation of Host diet to reduce gastrointestinal colonization by the opportunistic pathogen Candida albicans. mSphere. 2016;1(1). doi:10.1128/mSphere.00020-15. PMC4863630 27303684

[210] Zwolinska-Wcislo M, Brzozowski T, Budak A, Kwiecien S, Sliwowski Z, Drozdowicz D, Trojanowska D, Rudnicka-Sosin L, Mach T, Konturek SJ, et al. Effect of candida colonization on human ulcerative colitis and the healing of inflammatory changes of the colon in the experimental model of colitis ulcerosa. J Physiol And Pharmacol: An Off J Pol Physiol Soc. 2009;60(1):107–118. 19439813

[211] Marr KA, Seidel K, Slavin MA, Bowden RA, Schoch HG, Flowers MED, Corey L, Boeckh M. Prolonged fluconazole prophylaxis is associated with persistent protec tion against candidiasis-related death in allogeneic marrow transplant recipients: long-term follow-up of a randomized, placebo-controlled trial. Blood. 2000;96(6):2055–2061. doi:10.1182/blood.V96.6.2055. 10979947

[212] Wang W, Guo W, Li L, Fu Z, Liu W, Gao J, Shu Y, Xu Q, Sun Y, Gu Y. Gu YJBp: andrographolide reversed 5-FU resistance in human colorectal cancer by elevating BAX expression. Biochemical Pharmacol. 2016;121:8–17. doi:10.1016/j.bcp.2016.09.024. 27693317

[213] Seelbinder B, Chen J, Brunke S, Vazquez-Uribe R, Santhaman R, Meyer A-C, de Oliveira Lino FS, Chan K-F, Loos D, Imamovic L, et al. Antibiotics create a shift from mutualism to competition in human gut communities with a longer-lasting impact on fungi than bacteria. Microbiome. 2020;8(1):133. doi:10.1186/s40168-020-00899-6. 32919472 PMC7488854

[214] Heisel T, Johnson AJ, Gonia S, Dillon A, Skalla E, Haapala J, Jacobs KM, Nagel E, Pierce S, Fields D, et al. Bacterial, fungal, and interkingdom microbiome features of exclusively breastfeeding dyads are associated with infant age, antibiotic exposure, and birth mode. Front Microbiol. 2022;13:1050574. doi:10.3389/fmicb.2022.1050574. 36466688 PMC9714262

[215] Czerucka D, Piche T, Rampal P. Review article: yeast as probiotics–saccharomyces boulardii. Alimentary Pharmacol & Ther. 2007;26(6):767–778. doi:10.1111/j.1365-2036.2007.03442.x. 17767461

[216] Banik A, Halder SK, Ghosh C, Mondal KC. Fungal probiotics: opportunity, challenge, and prospects. Springer International Publishing; 2019.

[217] Guslandi M. Role of probiotics in Crohn’s disease and in Pouchitis. J Clin Gastroenterol. 2015;49(Supplement 1):S46–S49. doi:10.1097/MCG.0000000000000351. 26447964

[218] Sen S, Mansell TJ. Yeasts as probiotics: mechanisms, outcomes, and future potential. Fungal Genet And Biol: FG & B. 2020;137:103333. doi:10.1016/j.fgb.2020.103333. 31923554

[219] Guslandi M, Mezzi G, Sorghi M, Testoni PA. Saccharomyces boulardii in maintenance treatment of Crohn’s disease. Digestive Dis And Sci. 2000;45(7):1462–1464. doi:10.1023/A:1005588911207. 10961730

[220] Abbas Z, Yakoob J, Jafri W, Ahmad Z, Azam Z, Usman MW, Shamim S, Islam M. Cytokine and clinical response to saccharomyces boulardii therapy in diarrhea-dominant irritable bowel syndrome: a randomized trial. Eur J Gastroenterol & Hepatol. 2014;26(6):630–639. doi:10.1097/MEG.0000000000000094. 24722560

[221] Castagliuolo I, Riegler MF, Valenick L, LaMont JT, Pothoulakis C, Kozel TR. Saccharomyces boulardii protease inhibits the effects of Clostridium difficile toxins A and B in human colonic mucosa. Infect Immun. 1999;67(1):302–307. doi:10.1128/IAI.67.1.302-307.1999. 9864230 PMC96311

[222] Seed PC. The human mycobiome. Cold Spring harbor perspectives in medicine. Cold Spring Harbor Perspectives In Med. 2014;5(5):a019810. doi:10.1101/cshperspect.a019810. PMC4448585 25384764

[223] Qamar A, Aboudola S, Warny M, Michetti P, Pothoulakis C, LaMont JT, Kelly CP, Mansfield JM. Saccharomyces boulardii stimulates intestinal immunoglobulin a immune response to Clostridium difficile toxin a in mice. Infect Immun. 2001;69(4):2762–2765. doi:10.1128/IAI.69.4.2762-2765.2001. 11254650 PMC98222

[224] Kelesidis T, Pothoulakis C. Efficacy and safety of the probiotic saccharomyces boulardii for the prevention and therapy of gastrointestinal disorders. Ther Adv Gastroenterol. 2012;5(2):111–125. doi:10.1177/1756283X11428502. PMC3296087 22423260

[225] Pontier-Bres R, Munro P, Boyer L, Anty R, Imbert V, Terciolo C, André F, Rampal P, Lemichez E, Peyron JF, et al. Saccharomyces boulardii modifies Salmonella typhimurium traffic and host immune responses along the intestinal tract. PLOS ONE. 2014;9(8):e103069. doi:10.1371/journal.pone.0103069. 25118595 PMC4145484

[226] Gedek G. Adherence of Escherichia coli serogroup 0 157 and the Salmonella Typhimurium mutant DT 104 to the surface of saccharomyces boulardii. Mycoses. 1999;42(4):261–264. doi:10.1046/j.1439-0507.1999.00449.x. 10424093

[227] Saber A, Alipour B, Faghfoori Z, Yari Khosroushahi A. Cellular and molecular effects of yeast probiotics on cancer. Crit Rev Microbiol. 2017;43(1):96–115. doi:10.1080/1040841X.2016.1179622. 27561003

[228] Volman JJ, Ramakers JD, Plat J. Dietary modulation of immune function by β-glucans. Physiol & Behav. 2008;94(2):276–284. doi:10.1016/j.physbeh.2007.11.045. 18222501

[229] Tuman RW, Bilbo JT, Doisy RJ. Comparison and effects of natural and synthetic glucose tolerance fact or in normal and genetically diabetic mice. Diabetes. 1978;27(1):49–56. doi:10.2337/diab.27.1.49. 340311

[230] Kim R, An M, Lee H, Mehta A, Heo YJ, Kim K-M, Lee S-Y, Moon J, Kim ST, Min B-H, et al. Min B-HJCd: early tumor–immune microenvironmental remodeling and response to first-line fluoropyrimidine and platinum chemotherapy in advanced gastric cancer. Cancer Discov. 2022;12(4):984–1001. doi:10.1158/2159-8290.CD-21-0888. 34933901 PMC9387589

[231] Jayabalan R, Malbaša RV, Lončar ES, Vitas JS, Sathishkumar M. Sathishkumar MJCrifs, safety f: a review on kombucha tea—microbiology, composition, fermentation, beneficial effects, toxicity, and tea fungus. Compr Rev In Food Sci Food Saf. 2014;13(4):538–550. doi:10.1111/1541-4337.12073. 33412713

[232] Villarreal‐Soto SA, Beaufort S, Bouajila J, Souchard JP, Taillandier P. Taillandier PJJofs: understanding kombucha tea fermentation: a review. J Food Sci. 2018;83(3):580–588. doi:10.1111/1750-3841.14068. 29508944

[233] Jang CH, Oh J, Lim JS, Kim HJ, Kim J-S. Fermented soy products: beneficial potential in neurodegenerative diseases. Foods. 2021;10(3):636. doi:10.3390/foods10030636. 33803607 PMC8003083

[234] Shurtleff W, Aoyagi A. History of miso, soybean jiang (China), jang (Korea) and tauco (Indonesia)(200. (BC)-2009): Soyinfo Center; 2009.

[235] Hong S-B, Kim D-H, Samson RA. Aspergillus associated with Meju, a fermented soybean starting material for traditional soy sauce and soybean paste in Korea. Mycobiology. 2015;43(3):218–224. doi:10.5941/MYCO.2015.43.3.218. 26539037 PMC4630427

[236] Huo X, Li D, Wu F, Li S, Qiao Y, Wang C, Wang Y, Zhou C, Sun L, Luan Z, et al. Cultivated human intestinal fungus Candida metapsilosis M2006B attenuates colitis by secreting acyclic sesquiterpenoids as FXR agonists. Gut. 2022;71(11):2205. doi:10.1136/gutjnl-2021-325413. 35173042

[237] Kassam Z, Lee CH, Yuan Y, Hunt RH. Fecal microbiota transplantation for Clostridium difficile infection: systematic review and meta-analysis. The Am J Gastroenterol. 2013;108(4):500–508. doi:10.1038/ajg.2013.59. 23511459

[238] Debast SB, Bauer MP, Kuijper EJ. European society of clinical microbiology and infectious diseases: update of the treatment guidance document for Clostridium difficile infection. Clin Microbiol And Infect: The Off Publ Of The Eur Soc Of Clin Microbiol And Infect Dis. 2014;Suppl 20:1–26. doi:10.1111/1469-0691.12418. 24118601

[239] McDonald LC, Gerding DN, Johnson S, Bakken JS, Carroll KC, Coffin SE, Dubberke ER, Garey KW, Gould CV, Kelly C, et al. Clinical practice guidelines for clostridium difficile infection in adults and children: 2017 update by the infectious diseases society of America (IDSA) and society for healthcare epidemiology of America (SHEA). And Soc For Healthcare Epidemiology Of Am (SHEA) Clin Infect Dis: An Off Publ Of The Infect Dis Soc Of Am. 2018;66(7):987–994. doi:10.1093/cid/ciy149. 29562266

[240] Lam S, Bai X, Shkoporov AN, Park H, Wu X, Lan P, Zuo T. Roles of the gut virome and mycobiome in faecal microbiota transplanta tion. The Lancet Gastroenterol & Hepatol. 2022;7(5):472–484. doi:10.1016/S2468-1253(21)00303-4. 35276080

[241] Zuo T, Wong SH, Cheung CP, Lam K, Lui R, Cheung K, Zhang F, Tang W, Ching JYL, Jcy W, et al. Gut fungal dysbiosis correlates with reduced efficacy of fecal microbi ota transplantation in clostridium difficile infection. Nat Commun. 2018;9(1). doi:10.1038/s41467-018-06103-6. PMC6131390 30202057

[242] Kazemian N, Ramezankhani M, Sehgal A, Khalid FM, Kalkhoran AHZ, Narayan A, Wong GK, Kao D, Pakpour S. The trans-kingdom battle between donor and recipient gut microbiome influences fecal microbiota transplantation outcome. Sci Rep. 2020;10(1):18349. doi:10.1038/s41598-020-75162-x. 33110112 PMC7591866

[243] Leonardi I, Paramsothy S, Doron I, Semon A, Kaakoush NO, Clemente JC, Faith JJ, Borody TJ, Mitchell HM, Colombel JF, et al. Fungal trans-kingdom dynamics linked to responsiveness to fecal microbiota transplantation (FMT) therapy in ulcerative colitis. Cell Host & Microbe. 2020;27(5):823–829.e3. doi:10.1016/j.chom.2020.03.006. 32298656 PMC8647676

[244] Jalanka J, Mattila E, Jouhten H, Hartman J, de Vos Wm, Arkkila P, Satokari R, de Vos WM. Long-term effects on luminal and mucosal microbiota and commonly acquired taxa in faecal microbiota transplantation for recurrent Clostridium difficile infection. BMC Med. 2016;14(1):1–10. doi:10.1186/s12916-016-0698-z. 27724956 PMC5057499

[245] Strocchi A, Bond JH, Ellis C, Levitt MD. Colonic concentrations of hydrogen and methane following colonoscopic preparation with an oral lavage solution. Gastrointestinal Endoscopy. 1990;36(6):580–582. doi:10.1016/S0016-5107(90)71168-6. 2126250

[246] Wrzosek L, Ciocan D, Borentain P, Spatz M, Puchois V, Hugot C, Ferrere G, Mayeur C, Perlemuter G, Cassard AM. Transplantation of human microbiota into conventional mice durably reshapes the gut microbiota. Sci Rep. 2018;8(1):6854. doi:10.1038/s41598-018-25300-3. 29717179 PMC5931539

[247] Ji SK, Yan H, Jiang T, Guo CY, Liu JJ, Dong SZ, Yang KL, Wang YJ, Cao ZJ, Li SL. Preparing the gut with antibiotics enhances gut microbiota reprogramming efficiency by promoting xenomicrobiota colonization. Front Microbiol. 2017;8:1208. doi:10.3389/fmicb.2017.01208. 28702022 PMC5487471

[248] Bokoliya SC, Dorsett Y, Panier H, Zhou Y. Procedures for Fecal Microbiota Transplantation in Murine Microbiome Studies. Front Cell Infect Microbiol. 2021;11:711055. doi:10.3389/fcimb.2021.711055. 34621688 PMC8490673

[249] Danne C, Rolhion N, Sokol H. Recipient factors in faecal microbiota transplantation: one stool does not fit all. Nat Rev Gastroenterol Hepatol. 2021;18(7):503–513. doi:10.1038/s41575-021-00441-5. 33907321

[250] DeFilipp Z, Bloom PP, Torres SM, Mansour MK, Sater MRA, Huntley MH, Turbett S, Chung RT, Chen YB, Hohmann EL. Hohmann EL: drug-resistant E. coli bacteremia transmitted by fecal microbiota transplant. N Engl J Med. 2019;381(21):2043–2050. doi:10.1056/NEJMoa1910437. 31665575

